# Breaking the Brønsted–Evans–Polanyi Relation with Dual-Metal Sites

**DOI:** 10.1021/acs.jpclett.5c02446

**Published:** 2025-10-23

**Authors:** Yiming Chen, Haohong Song, Zili Wu, De-en Jiang

**Affiliations:** † Department of Chemical and Biomolecular Engineering, 5718 Vanderbilt University, Nashville, Tennessee 37235, United States; ‡ Interdisciplinary Materials Science, Vanderbilt University, Nashville, Tennessee 37235, United States; § Chemical Sciences Division, 6146 Oak Ridge National Laboratory, Oak Ridge, Tennessee 37831, United States; ∥ Center for Nanophase Materials Sciences, Oak Ridge National Laboratory, Oak Ridge, Tennessee 37831, United States

## Abstract

Linear scaling relationships impose inherent limitations on catalyst activity; the Brønsted–Evans–Polanyi (BEP) relation, which correlates activation and reaction energies, is a prominent example. Here we report a dual-metal site catalyst (DMSC) on ceria that breaks the BEP relation for C–C coupling of methyl intermediates—an elementary step in methane coupling to form ethane. The DMSC structure on CeO_2_(111) was discovered by density-functional theory (DFT) structural exploration and confirmed to be stable via ab initio thermodynamics and ab initio molecular dynamics. Homonuclear and heteronuclear DMSCs of Ni, Pd, Pt, Fe, Ru, Os, Co, Rh, and Ir (45 pairs in total) were examined for methyl affinity and methyl–methyl coupling activation energy. We found that many heteronuclear DMSCs break the BEP linear scaling due to a mixed low-affinity/high-affinity coadsorption of the two methyl groups, decoupling the step responsible for the activation energy (*E*_a_) at the low-affinity site from the overall reaction energy (Δ*E*) determined by both sites. This mechanism of breaking the BEP relationship via the DMSCs offers a catalyst design principle for C–C coupling reactions.

## Graphical abstract

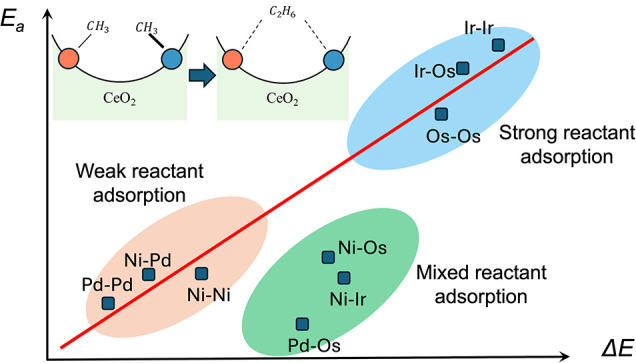

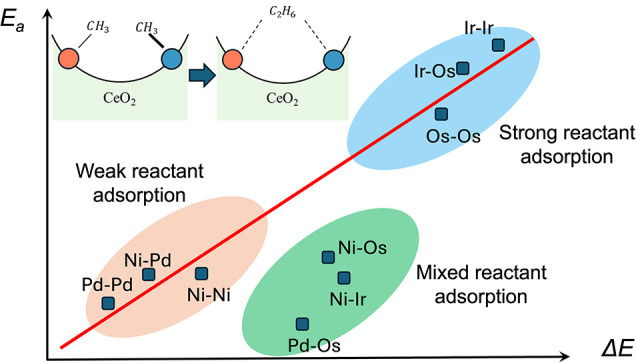

Linear scaling relationships (LSRs) in catalysis characterize the linear dependencies among key reaction descriptors, such as activation energy, adsorption energy of reactants, and reaction energy.^1–4^ Among them, the Brønsted–Evans–Polanyi (BEP) relation states the positive linear correlation between activation energy and reaction energy, as shown by the linear scaling line in Figure 1a.^5^ These LSRs facilitate the rational design of catalysts,^6–8^ as the kinetics of catalysis can be evaluated through screening the thermodynamic parameters like reaction energy. While the BEP relation and other LSRs guide the catalyst design, they also imply an intrinsic limitation on catalysts’ reactivity. The volcano plot of the catalyst’s activity, introduced first by Sabatier,^9^ illustrates that catalysts exhibit the best catalytic performance when reaching a medium level of affinity, where affinity should be strong enough to adsorb reactants and enable reactions, while weak enough to desorb the product from the surface of the catalyst to sustain catalysis. In addition to the conventional approach of tuning elements of catalysts to achieve the peak on the volcano plot,^10,11^ efforts have been made to design catalysts beyond the volcano plot by breaking the LSRs.^12,13^

**Figure 1:**
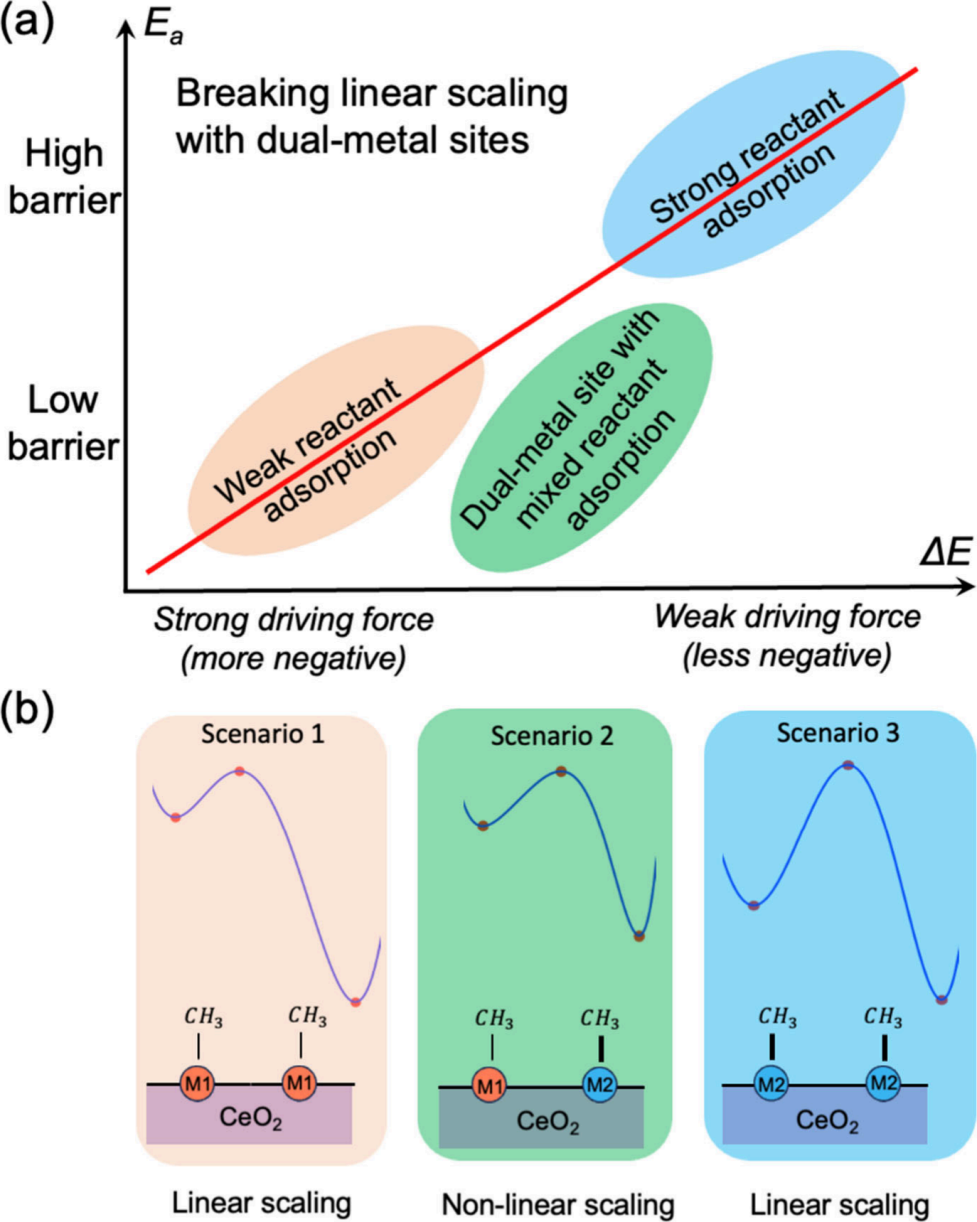
Idea of using dual-metal-site catalysts (DMSCs) to break the Brønsted–Evans–Polanyi linear scaling relationship for the methyl–methyl coupling reaction: (a) Activation energy vs reaction energy—the red line is the linear scaling relation; (b) three scenarios of reaction energy profiles on ceria-supported DMSCs where the nonlinear scaling originates from the two metal sites having different affinities for methyl adsorption.

Dual-metal-site catalysts (DMSCs) have attracted great interest recently in catalysis due to the synergistic effects between two metal sites and many other benefits.^14–16^ DMSCs are different from conventional bifunctional catalysts in that they are specifically referred to as two sites composed of single atoms, while conventional bifunctional catalysts typically comprise two different particles or one particle decorated with another site of single atom or clusters. Studies have revealed the capability of DMSCs to decouple the adsorption energies of reactants from those of products, due to the presence of metal sites with varying affinities toward different elements.^17–19^ However, the power of DMSCs to break the BEP relation, i.e., to decouple the activation energy from the reaction energy, remains to be explored. Moreover, similar to single-atom catalysts (SACs), DMSCs are prone to sintering and aggregation at high temperatures,^19–21^ which leads to the loss of functional configurations and thus a substantial reduction in catalysts’ activity. Hence, high stability is desirable for a DMSC.

Herein, we introduce a stable and unique DMSC structure supported on the (111) surface of ceria. Nine transition metals in Groups 8–10 (i.e., Group VIII) were chosen to construct both homonuclear and heteronuclear DMSC catalysts (45 in total), as these elements in dispersed metal sites^2,16,22,23^ are widely used in catalysis.^15,24,25^ We use the coupling of two methyl groups, one of the key steps for oxidative coupling of methane (OCM),^26,27^ as a probe reaction to demonstrate the capability of such ceria-supported DMSCs to break the BEP relation. Although at typical thermocatalytic OCM temperatures (∼700 °C) the methyl groups generated on catalyst surfaces desorb and couple as radicals in gas phase, there is growing interest in exploring lower temperature^28^ and nonthermal^29^ surface-coupling pathways as well as nonoxidative coupling of methane,^27,30^ where our proposed DMSCs might be useful.

The key idea of using the DMSCs to break the BEP relation for the methyl–methyl coupling reaction is illustrated in Figure 1: when both metal sites have low affinity for methyl, the coupling reaction has a low barrier and strong driving force (Scenario 1; pink); when both metal sites have high affinity for methyl, the coupling reaction has a high barrier and weak driving force (Scenario 3; blue). Both Scenarios 1 and 3 conform to the BEP relation. In contrast, when the two metal sites have different or mixed affinities for methyl, the coupling reaction has a low barrier and moderate driving force (scenario 2; green), thereby decoupling the activation energy from the overall reaction energy and breaking the BEP relation. Below we show our computational proof-of-concept to demonstrate this idea.

To create stable ceria-supported DMSCs for Group VIII metals, we chose the CeO_2_(111) surface as the base and explored substitutional surface doping by replacing two surface [CeO]^2+^ units with two Group VIII metal dications. The detailed strategy to construct the DMSCs is discussed in the Supporting Information (Note 1 and Figures S1–S3). The full set of configurations for the Pd–Ni DMSCs is provided in Table S1 as an example for heteronuclear DMSCs. Different from previously reported DMSC structures on CeO_2_(111),^31,32^ the most stable configuration found is shown in Figure 2a where the two metal sites create a surface pit on CeO_2_(111) (see Figure S4 for the local environment of the concave DMSC and the movie file in SI for a three-dimensional view of the supercell; we name this concave DMSC structure configuration A). The thermal stability of this DMSC structure was confirmed via ab initio molecular dynamics (AIMD) simulations at 1000 K for 10 ps using Pd–Ni and Pd–Pd DMSCs as representative examples, which showed the structure to be highly stable (Figures S5–S6). This structure was then used as a template to host the 36 heteronuclear DMSCs and 9 homonuclear DMSCs among Fe, Co, Ni, Ru, Rh, Pd, Os, Ir, and Pt. To further evaluate the stability of the DMSCs against sintering and oxidation, we performed ab initio thermodynamics analysis (see Note 2 in SI for details) following two previous studies.^33,34^ We compared configuration A that we found in this work (Figure 2a) with DMSCs from the literature and a typical +4 oxidation-state configuration where the two metal atoms simply replace two Ce^4+^ on the surface (Figure S7). As shown in Figure S8, configuration A remains the most stable structure at 600 °C across a range of O_2_ pressures (pO_2_). We further examined the stability of configuration A against sintering to large metal nanoparticles or oxidation to the +4 state: stability diagrams in Figure S9 show that configuration A is stable in phase fields of certain temperature and pO_2_ ranges and the areas of the phase fields are especially large for group 9 and group 10 elements.

**Figure 2:**
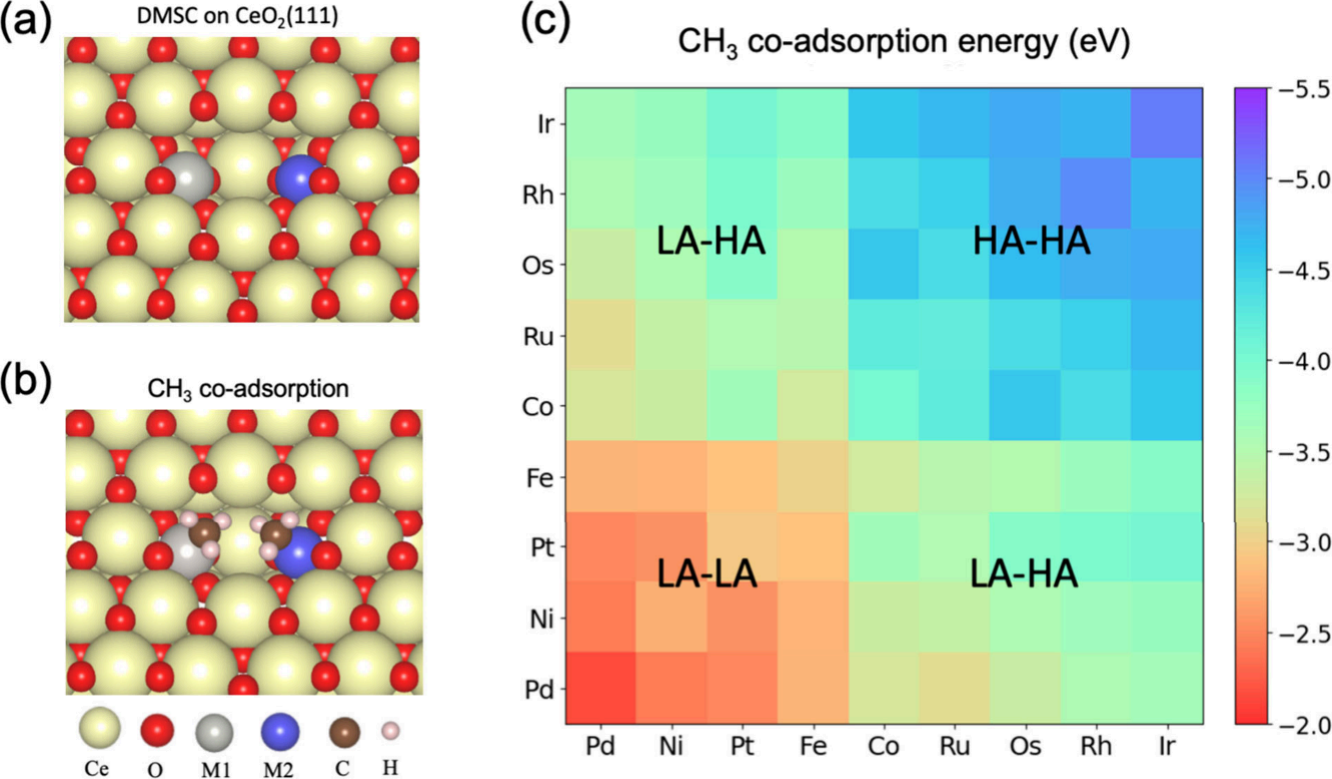
(a) The DMSC structure on CeO_2_(111) (see the movie in SI for a 3D view). (b) Co-adsorption of two methyl groups on the ceria-supported DMSC. (c) Heat map of coadsorption energies of two methyl groups on the 45 ceria-supported DMSCs [*E*_ad_ = *E*(2CH_3_-DMSC) – *E*(DMSC) – 2*E*(CH_3_)].

We next examined the coadsorption of two methyl groups on the DMSCs: as shown in Figure 2b, the two methyl groups can be comfortably accommodated in the surface pit, tilting toward each other. Figure 2c shows the methyl coadsorption energies [*E*_ad_ = *E*(2CH_3_-DMSC) – E­(DMSC) – 2*E*(CH_3_)] as a heatmap for all the 45 DMSCs examined: Ir–Ir DMSC exhibits the strongest affinity (*E*_ad_ = −5.08 eV), while Pd–Pd DMSC shows the weakest affinity (*E*_ad_ = −2.14 eV). Based on the whole map, we can categorize the nine Group VIII elements into two groups based on the binding strength with the methyl group: high-affinity (HA) elements (Ir, Rh, Os, Ru, Co) and low-affinity (LA) elements (Fe, Pt, Ni, Pd). We can further categorize the 45 DMSCs in Figure 2c as three groups: LA-LA where both metals have weak CH_3_ adsorption (lower-left corner); HA-HA where both metals have strong CH_3_ adsorption (upper-right corner); and LA-HA where one metal has weak and the other has strong CH_3_ adsorption (off-diagonal corners).

From the coadsorbed states of two methyl groups on the DMSCs, we then determined the transition states and activation energies (*E*_a_) for the methyl–methyl coupling reaction to form an adsorbed ethane molecule (Figure 3a). The reaction energy (Δ*E*) represents the energy of the adsorbed ethane relative to the coadsorption state of two CH_3_ groups on the DMSC. Figure 3b plots *E*_a_ vs Δ*E* for all 45 DMSCs. Indeed, one can see three regions that correspond to the three scenarios in Figure 1 and the three categories of methyl coadsorption in Figure 2c. All homonuclear DMSCs are along the linear-scaling line, as they belong to either the LA-LA category (scenario 1: low barrier, strong driving force) or the HA-HA category (scenario 3: high barrier, weak driving force). The heteronuclear DMSCs can be LA-LA (∼20%), HA-HA (∼30%), or LA-HA (50%; scenario 2: low barrier, moderate driving force). More importantly, many DMSCs in the LA-HA category break the BEP scaling relation (Figure 3b, green group), thereby proving the concept that we have envisioned in Figure 1.

**Figure 3:**
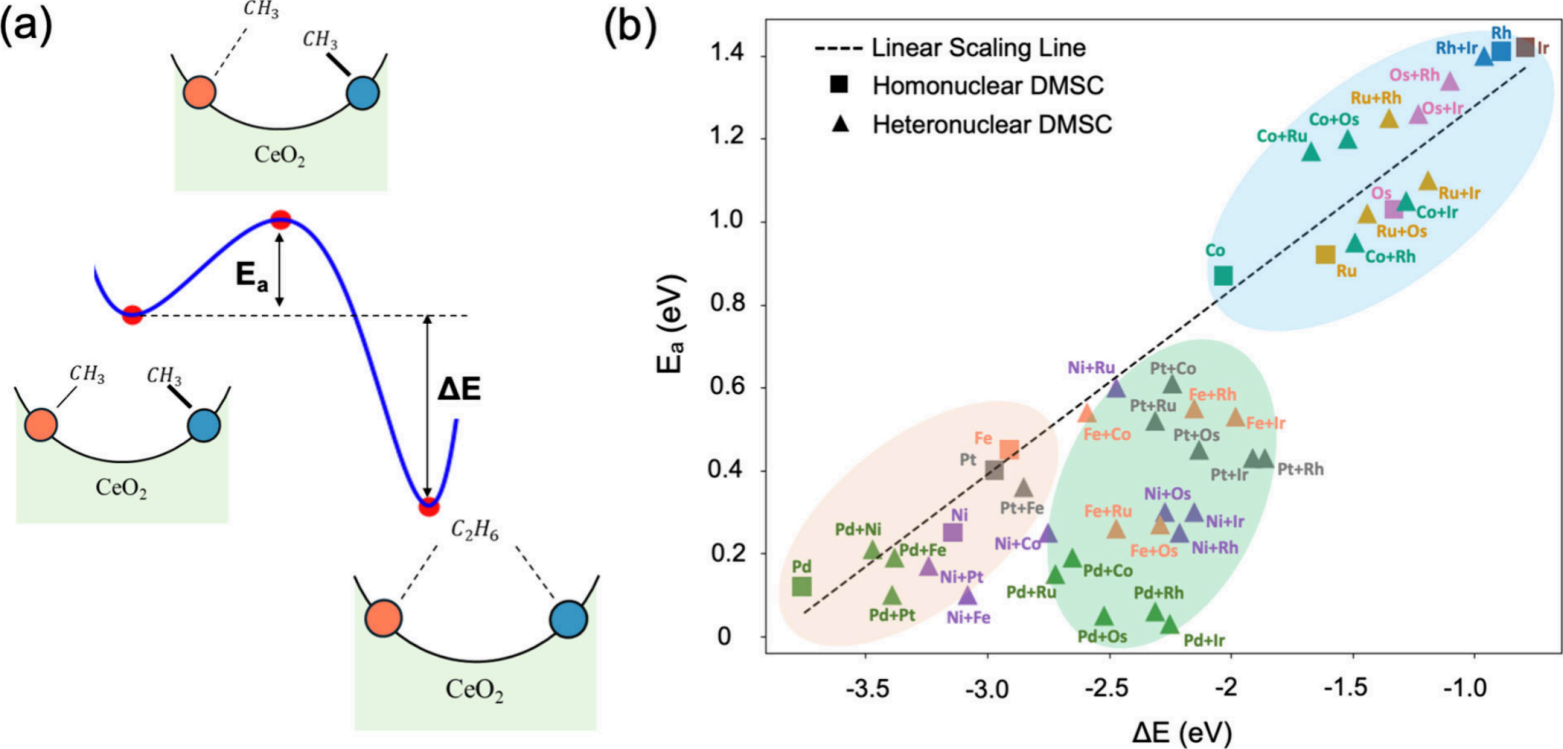
(a) Schematic of CH_3_–CH_3_ coupling reaction on a ceria-supported DMSC. (b) *E*_a_ vs Δ*E* of CH_3_–CH_3_ coupling reaction on the 45 ceria-supported DMSCs.

Figure 3b shows that pairing an earth-abundant metal such as Ni with a noble metal such as Ir can have the combined benefit of high activity (due to lower activation energy) and moderate heat release (due to a less negative enthalpy change). Figure 4 illustrates the detailed reaction process of methyl–methyl coupling on the Ni–Ir/CeO_2_(111) DMSC as an example (others are provided in Figures S10–S11 and Table S2). One can see that one methyl group first detaches from the Ni site with an *E*_a_ of 0.29 eV (Figure 4b), then goes through a planar-geometry, shallow intermediate on the surface (Figure 4c), and next attacks the other methyl strongly adsorbed on the Ir site (Figure 4d), leading to CH_3_CH_3_ formation (Figure 4e). The whole process takes place in the surface pit of the DMSC. In contrast, the desorption of the CH_3_ group from the Ni site to the gas phase is much higher in energy (in other words, the Eley–Rideal mechanism is unlikely in this case).

**Figure 4:**
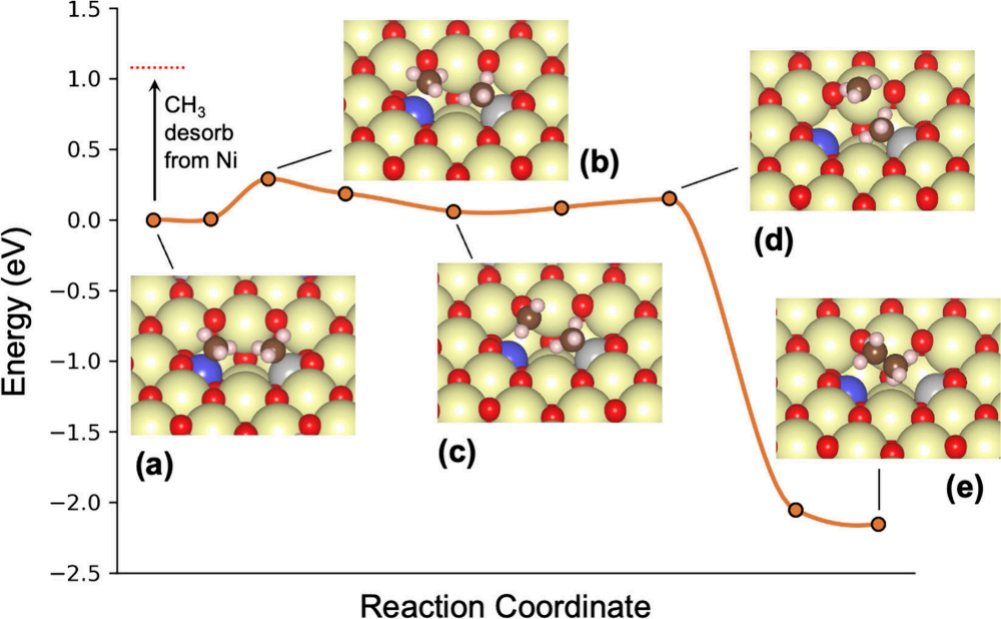
Minimum energy path of methyl–methyl coupling on the Ni-Ir/CeO_2_(111) DMSC. The dotted red line indicates the energy needed for CH_3_ to desorb from the Ni site to the gas phase as a radical. (Ni, blue; Ir, gray).

Figure 4 confirms the concept of decoupling the activation energy from the reaction energy at the DMSC site. In the rate-limiting step of the first methyl detachment/activation (Figure 4b), it takes place at the LA site (Ni in this case), so the activation energy is primarily governed by the energy required to break the Ni-CH_3_ bond at this LA site. The subsequent, second methyl detachment/activation from the HA site (Ir in this case) and the following methyl–methyl coupling are greatly facilitated by the approaching first methyl group, leading to a lower activation energy (Figure 4d) than the first one (Figure 4b). On the other hand, the overall reaction energy is determined by the total energy of dissociating both methyl groups from the two metal sites and the formation of the CH_3_–CH_3_ bond, which is the result of the combined effects of *both* LA and HA sites. Because the rate-limiting step is the metal-methyl bonding breaking at the LA site while the overall reaction energy is governed by the metal-methyl bonding breakings at both the LA and HA sites, the linear scaling is broken. Hence, our work shows that there is great opportunity in enabling surface CH_3_–CH_3_ coupling by using the DMSCs that break the BEP relation.

Our study uses methyl coupling as an example to illustrate a decoupling strategy arising from intrinsic differences in adsorption strengths between two sites in the DMSCs, which in principle could extend to other C–C coupling reactions and broader processes involving dual active sites. However, its broader applicability may depend on the electronic and structural properties of the reactants and sites, requiring further study to test its universality. In addition, DMSCs like SACs are prone to sintering; recent studies show that support engineering (such as via Ti doping^35^ or steaming to create surface −OH groups^36^) can greatly enhance the SAC stability on the ceria support. Such strategies can also be leveraged for stabilizing DMSCs on ceria.

In summary, our study demonstrates that DMSCs on ceria can overcome the intrinsic limitations imposed by LSRs, particularly the BEP relation, in the context of C–C coupling of methyl intermediates. Through extensive DFT-based structural exploration, ab initio thermodynamics analysis, and AIMD validation, we identified stable DMSCs on CeO_2_(111) surfaces. Among the 45 homonuclear and heteronuclear DMSCs evaluated, many heteronuclear combinations were found to break the BEP relation by enabling asymmetric coadsorption of methyl species—where one site governs the activation energy and both sites dictate the reaction energy. This decoupling mechanism offers a generalizable design strategy for developing catalysts capable of an enhanced surface C–C coupling performance beyond traditional scaling constraints.

## Computational Methods

Density functional theory (DFT) calculations were performed using the Vienna Ab initio Simulation Package (VASP).^37,38^ The Perdew–Burke–Ernzerhof (PBE) functional of generalized-gradient approximation (GGA) was employed for electron exchange and correlation.^39^ The electron–core interaction was modeled using the projector-augmented wave method (PAW).^40,41^ The van der Waals dispersion force was included by using the zero damping DFT-D3 method of Grimme.^42^ The on-site Coulomb interaction was considered by employing the DFT+U method by Dudarev et al. A U value of 4.5 eV was selected for Ce 4f localized electrons based on previous studies.^15,27,31^ Plane-wave kinetic energy cutoff was set to 500 eV to treat the valence electrons. For constructing the dual-metal-site catalyst supported on CeO_2_(111), we selected a periodic slab with a (3 × 3) supercell with a 13 Å vacuum space. The CeO_2_(111) slab model consists of nine layers with Ce atoms distributed across three layers and O atoms distributed across six layers. The bottom four layers were fixed during DFT calculations, and only the atoms in the top five layers were allowed to relax. The Brillouin zone was sampled using a (2 × 2 × 1) k-mesh. Transition states were identified using the climbing-image nudged elastic band method and verified by frequency analysis.^43^

## Supplementary Material







## Notes

Detailed procedures and configurations in exploring the DMSC on CeO_2_(111); details of ab initio thermodynamics analysis and stability comparisons and diagrams; structural details and AIMD simulations of the most stable configuration; initial, transition, and final states and energetics of CH_3_–CH_3_ coupling on DMSCs; coordinates of optimized structures (PDF)
Movie file showing a three-dimensional view of the supercell (MOV)
Transparent Peer Review report available (PDF)
