# Supplementary material for: Breaking the Brønsted–Evans–Polanyi Relation with Dual-Metal Sites
Source: J Phys Chem Lett. 2025 Oct 23;16(43):11302–7. doi: 10.1021/acs.jpclett.5c02446 (PMC12581165; doi:10.1021/acs.jpclett.5c02446)
Supplement: Supplementary file 1 [file jz5c02446_si_001.pdf]

Supporting Information

Breaking the Brønsted−Evans−Polanyi Relation with Dual-Metal Sites

Yiming Chen[a], Haohong Song[b], Zili Wu[c,d], De-en Jiang[a,*]

[a] Department of Chemical and Biomolecular Engineering, Vanderbilt University, Nashville, Tennessee, 37235, United States
[b] Interdisciplinary Materials Science, Vanderbilt University, Nashville, Tennessee, 37235, United States
[c] Chemical Sciences Division, Oak Ridge National Laboratory, Oak Ridge, Tennessee 37831, United States
[d] Center for Nanophase Materials Sciences, Oak Ridge National Laboratory, Oak Ridge, Tennessee 37831, United States

* Corresponding author. E-mail: de-en.jiang@vanderbilt.edu

Note 1: Construction of the DMSC on CeO_2(111)

To create the dual TM^{2+} sites on CeO_2(111), we replace two neighboring $[CeO]^{2+}$ groups on the CeO_2(111) surface by two TM^{2+} ions. As shown in **Figure S1**, surrounding each Ce atom, there are six different positions of O atoms in the first and second surface layers. This yields many possible combinations in replacing two neighboring $[CeO]^{2+}$ groups by two TM^{2+} ions. Using Pd-Ni as an example (That is, Pd replaces one $[CeO]^{2+}$ group and Ni replaces the other), we have explored all of them (33 in total) and list the optimized geometry and energies in **Table S1**. The relative energies, ranging over 3 eV, are also plotted in **Figure S2**. The substitution configuration leading to the most stable DMSC (#33 in Figure S2; also denoted as **configuration A**) is shown in **Figure S3**: after geometry optimization and some surface reconstruction, the DMSC has a symmetric, concave pit-like structure which can be more clearly seen in **Figure S4**. This structure is highly stable, as evidenced by the stable, constant local geometry and minimal structural change after AIMD simulation at 1000 K for 10 ps (**Figure S5**). AIMD simulations were performed within the canonical (NVT) ensemble using Nosé–Hoover thermostats,[1,2] with a time step of 1 femtosecond (fs) and a total simulation time of 10 picoseconds (ps). We further found that this structure is also highly stable for other combinations of DMSCs, either homonuclear or heteronuclear ones. **Figure S6** shows the

AIMD simulation result of the homonuclear Pd DMSC of the same structure, which again confirms the minimal structural change at 1000 K.

Note 2: Ab initio thermodynamics

The system free energy for three configurations of DMSCs were calculated using fully oxidized systems, namely, the bulk CeO_2 and MO_2 (M = Ir, Rh, Pt, and Ni), as references. The expressions for the free energy of each configuration are shown below:

$$G_{Config.A} = E_{slab} + 2\mu_{MO_2} + E_{O_2} - E_{HS-2CeO} - 2\mu_{CeO_2} + \mu_{O_2}$$

$$G_{Config.B} = E_{slab} + 2\mu_{MO_2} + E_{O_2} - E_{NHS-2CeO} - 2\mu_{CeO_2} + \mu_{O_2}$$

$$G_{Config.C} = E_{slab} + 2\mu_{MO_2} + E_{O_2} - E_{1Ce} - \mu_{CeO_2} + \mu_{O_2}$$

where $E_{Config.x}$ $(x = A, B, or\ C)$, E_{slab}, and E_{O_2} are the DFT-calculated energies of the different DMSC configurations, pristine $CeO_2(111)$ surface and gaseous oxygen, respectively. μ_{MO_2}, μ_{CeO_2} represent the chemical potentials of bulk MO_2 and CeO_2, obtained also from the DFT calculations at 0K. Entropy and enthalpy corrections are neglected, consistent with previous ab initio thermodynamic studies of Pd atom on ceria surface.[3]

The chemical potential of gaseous oxygen, μ_{O_2}, was calculated as a function of pressure and temperature according to:

$$\mu_{O_2}(T, P) = \mu_{O_2}(T, P^0) + RTln(\frac{P_{O_2}}{P^0})$$

$$= \left[H_{O_2}(T, P^0) - H_{O_2}(0K, P^0)\right] - T\left[S_{O_2}(T, P^0) - S_{O_2}(0K, P^0)\right] + RTln(\frac{P_{O_2}}{P^0})$$

where the enthalpy $H_{O_2}(T, P^0)$ and entropy $S_{O_2}(T, P^0)$ of gaseous oxygen were obtained from the NIST-JANAF thermochemical tables.[4]

The relative stability of various oxidation states in the phase diagrams (**Figure S9**) was evaluated following the approach proposed by Reuter and Scheffle.[5] The stability of metal nanoparticles was estimated using the nominal neutral metal site model, in which the nearest oxygen is removed from the 2+ metal site, as proposed by Su, et al.[6] The relative free energy difference of metal nanoparticles (NP) and 4+ DMSCs (Config. D) with respect to 2+ DMSCs is determined as follows:

$$\Delta G_{NP} = (E_{NP} - E_{Config.A} + E_{O_2}) + \mu_{O_2}(T, P)$$

$$\Delta G_{Config.D} = \left(E_{Config.D} - E_{Config.A} - E_{O_2}\right) - \mu_{O_2}(T, P)$$

where $E_{Config.A}$, E_{NP}, and $E_{Config.D}$ are the DFT-calculated energies.

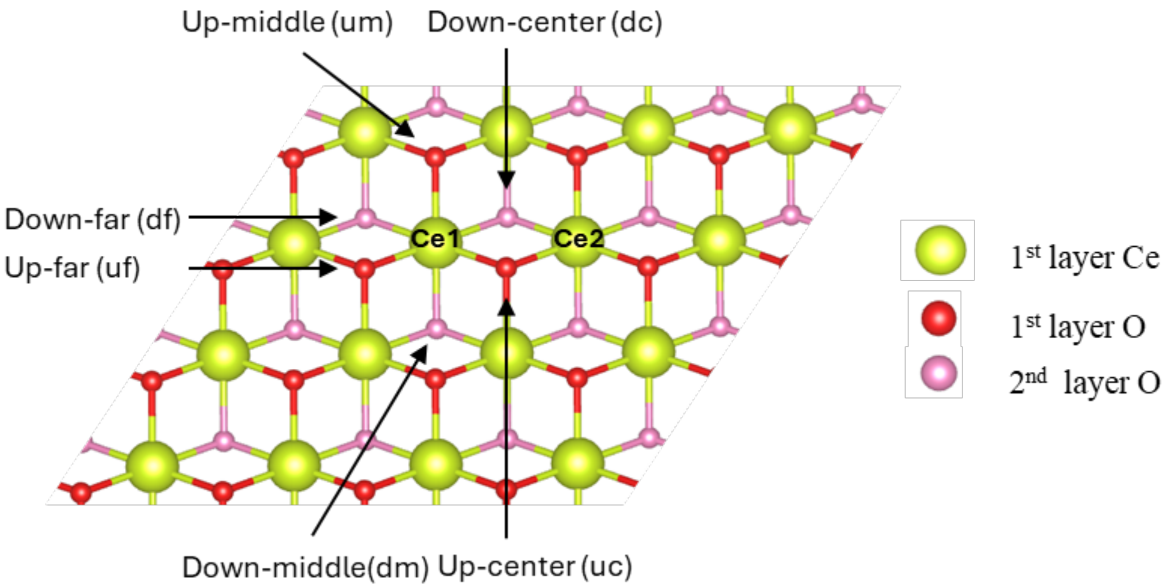

Figure S1. Replacing two neighboring surface $[CeO]^{2+}$ units at Ce1 and Ce2 by two transition-metal dications (TM^{2+}) to create a dual-metal-site catalyst (DMSC) on $CeO_2(111)$. The different O atoms around Ce1 and Ce2 are labelled as potential sites for removal with Ce ions. Top view.

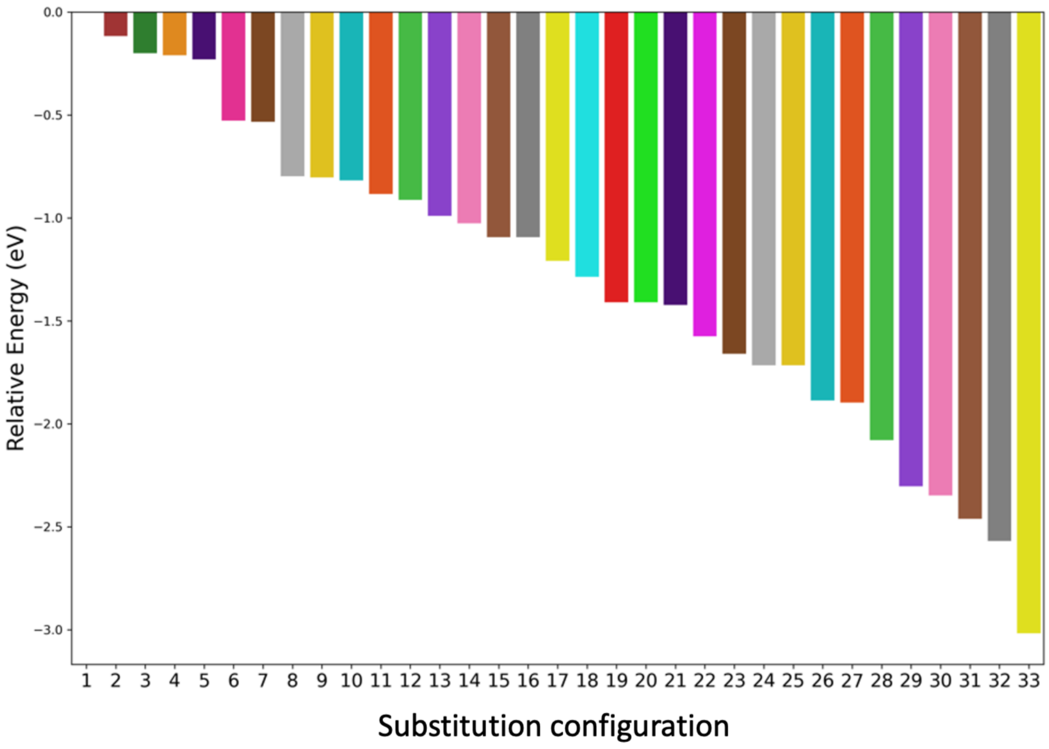

Figure S2. Relative energies for all 33 substitution configurations of Pd-Ni DMSCs on $CeO_2(111)$ after DFT geometry optimization (see the optimized structures in Table S1).

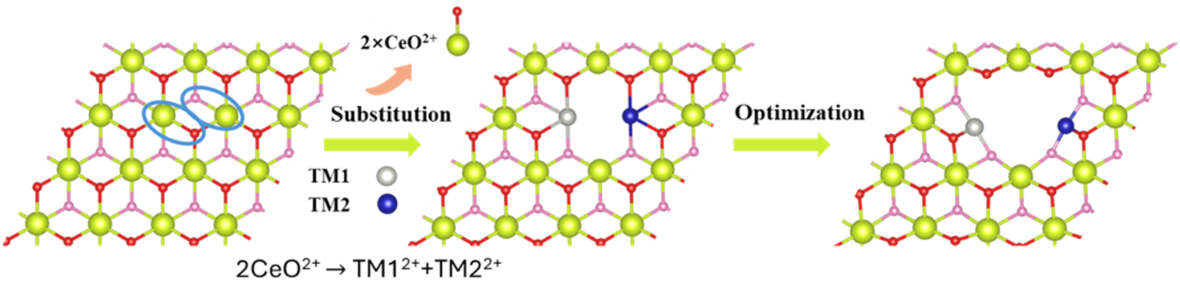

Figure S3. The substitution mode that leads to the most stable DMSC on $CeO_2(111)$: it has a concave, pit-like shape after geometry optimization (top view); this structure is denoted as configuration A.

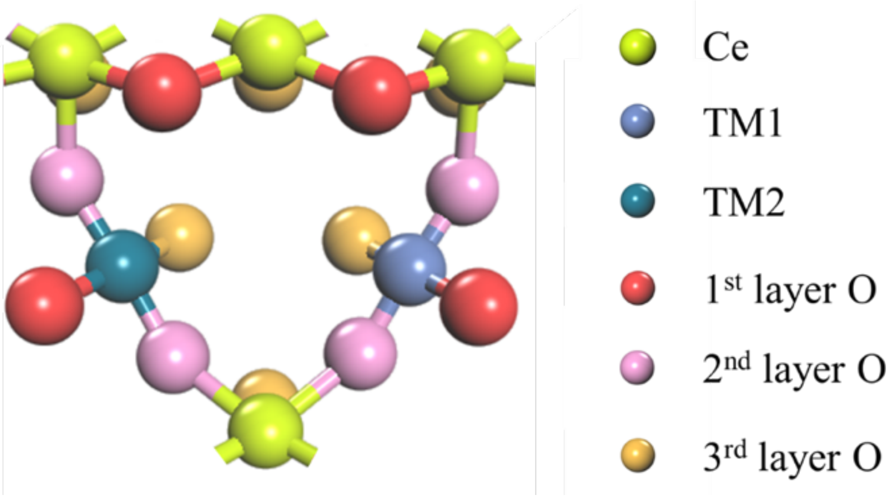

Figure S4. The local environment (top view) of the two TM sites in configuration A of the DMSC structure on $CeO_2(111)$.

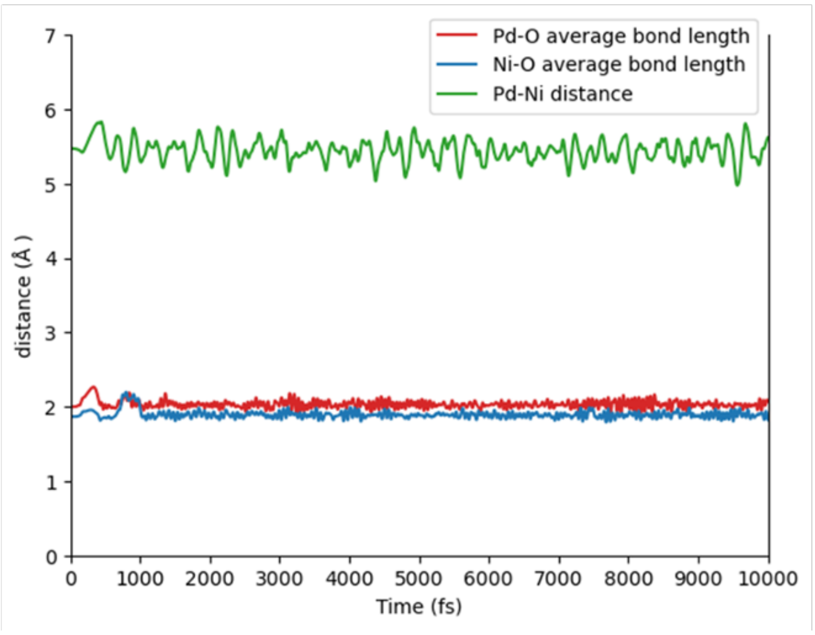

Figure S5. AIMD simulation for the Pd-Ni DMSC in configuration A at 1000K for 10 ps: Variation of the distance of Pd-Ni dual metal sites and the average bond lengths of the Pd-O bonds and Ni-O bonds.

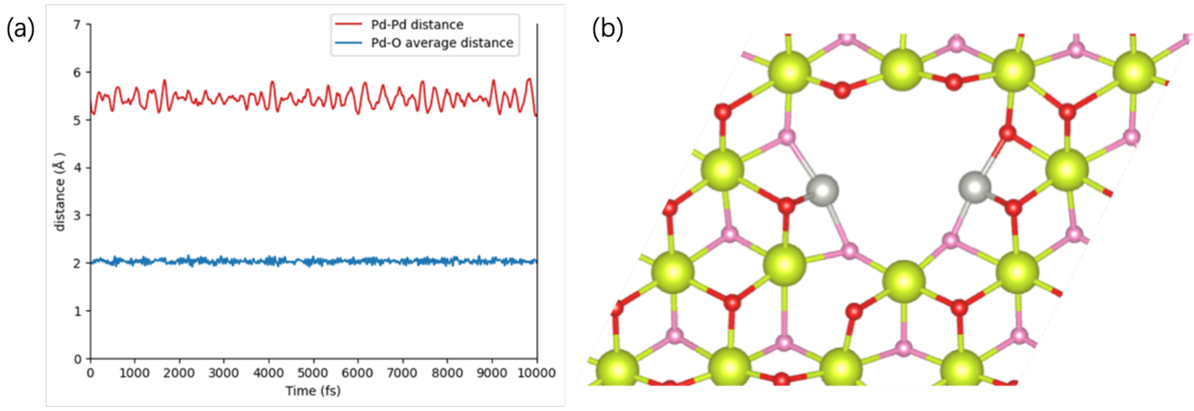

Figure S6. AIMD simulation for the Pd-Pd homonuclear DMSC in configuration A: (a) Variation of the distance of Pd-Pd dual metal sites and the average bond lengths of the Pd-O bonds; (b) Structure after AIMD simulation.

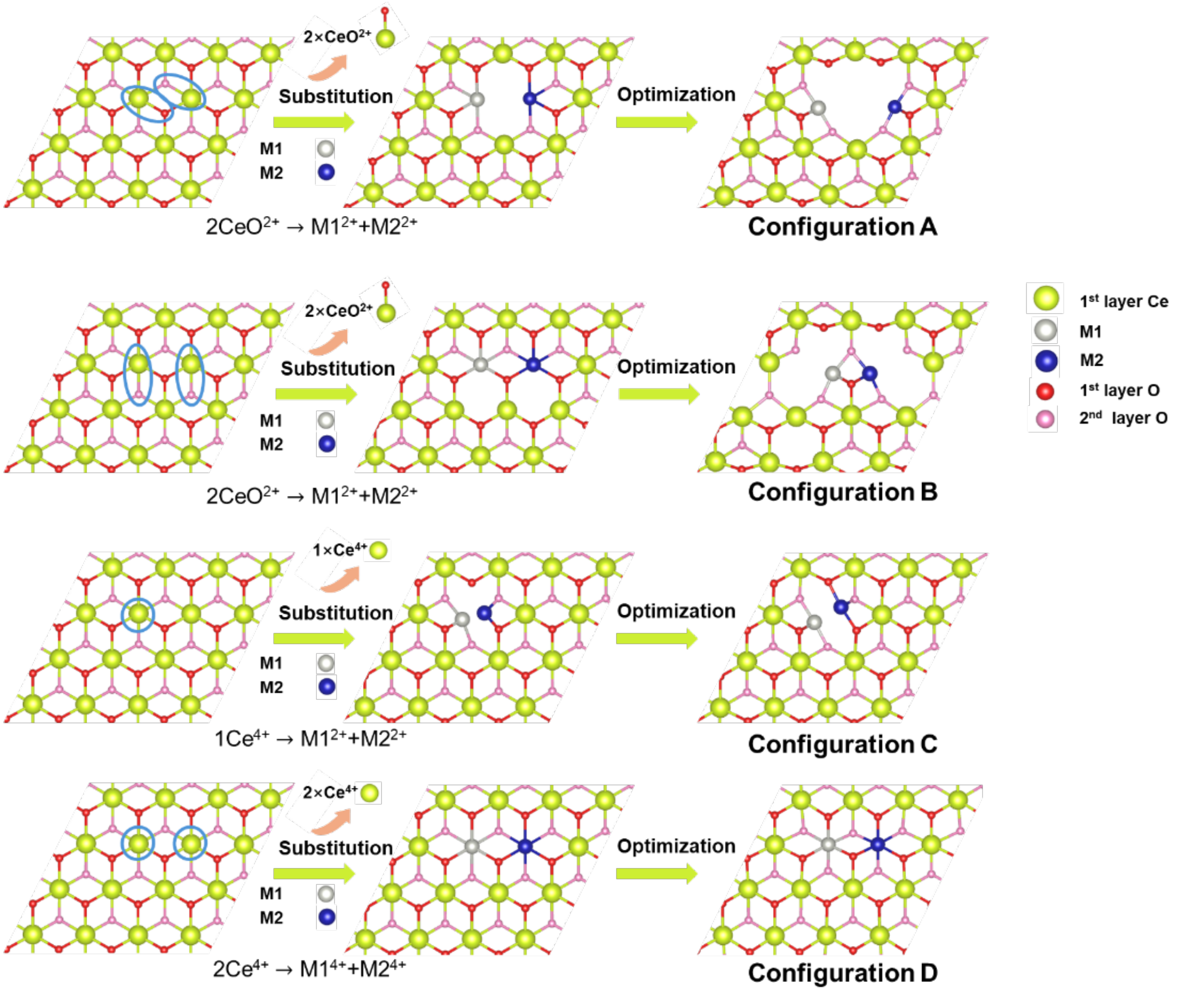

Figure S7. Four typical configurations of the DMSCs on $CeO_2(111)$: M1 and M2 in a 2+ nominal oxidation state in configurations A, B, and C but 4+ in configuration D. Configuration A is the one used in the text that shows the linear-scaling-breaking behavior; Configuration B is from ref. 31 in the text; Configuration C is from ref. 32 in the text; Configuration D is created from replacing two surface Ce^{4+} with $M1^{4+}$ and $M2^{4+}$.

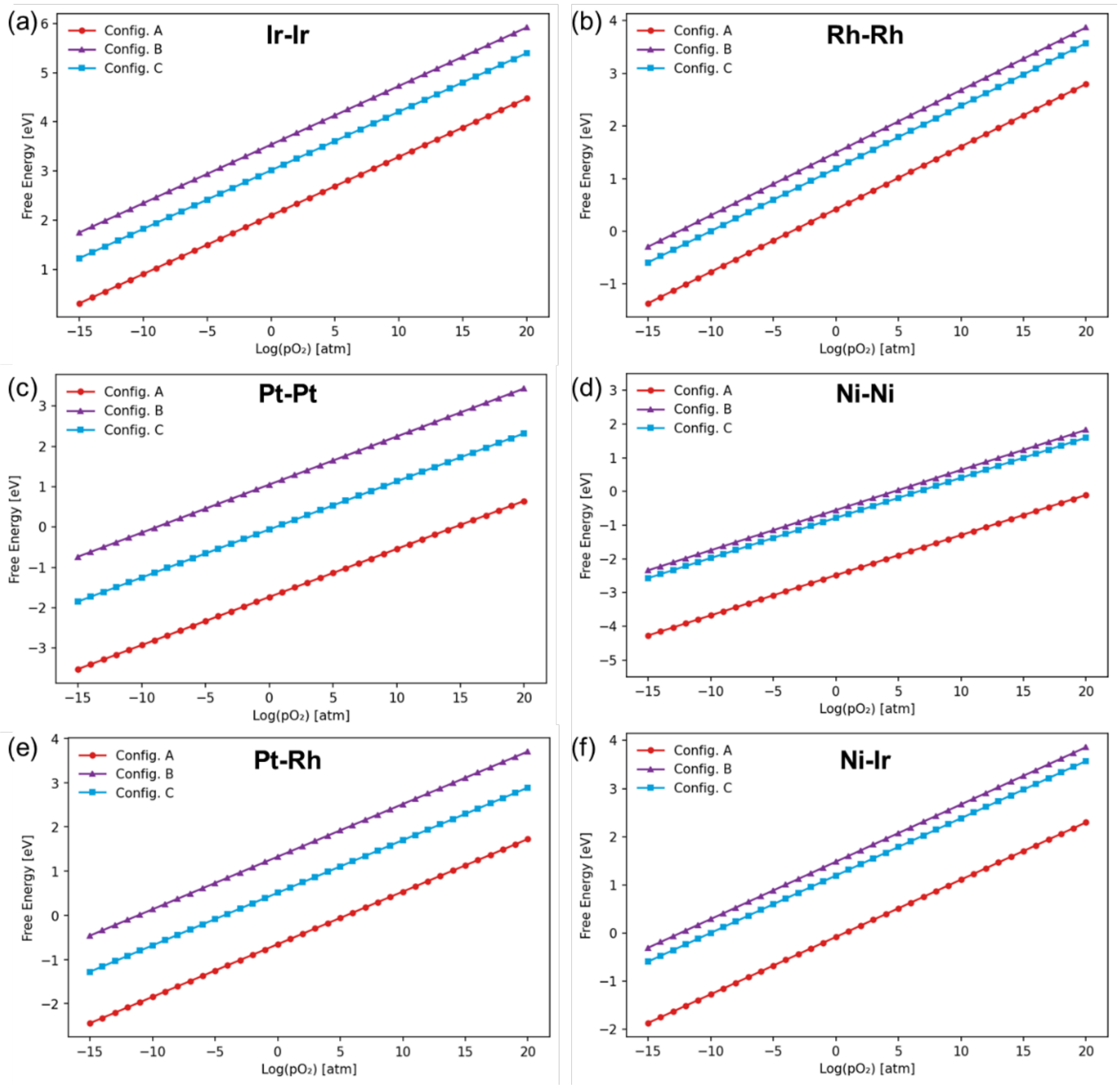

Figure S8. Relative stability of configurations A, B, and C for Ir-Ir, Rh-Rh, Pt-Pt, Ni-Ni, Pt-Rh, Ni-Ir DMSCs at 600 ºC.

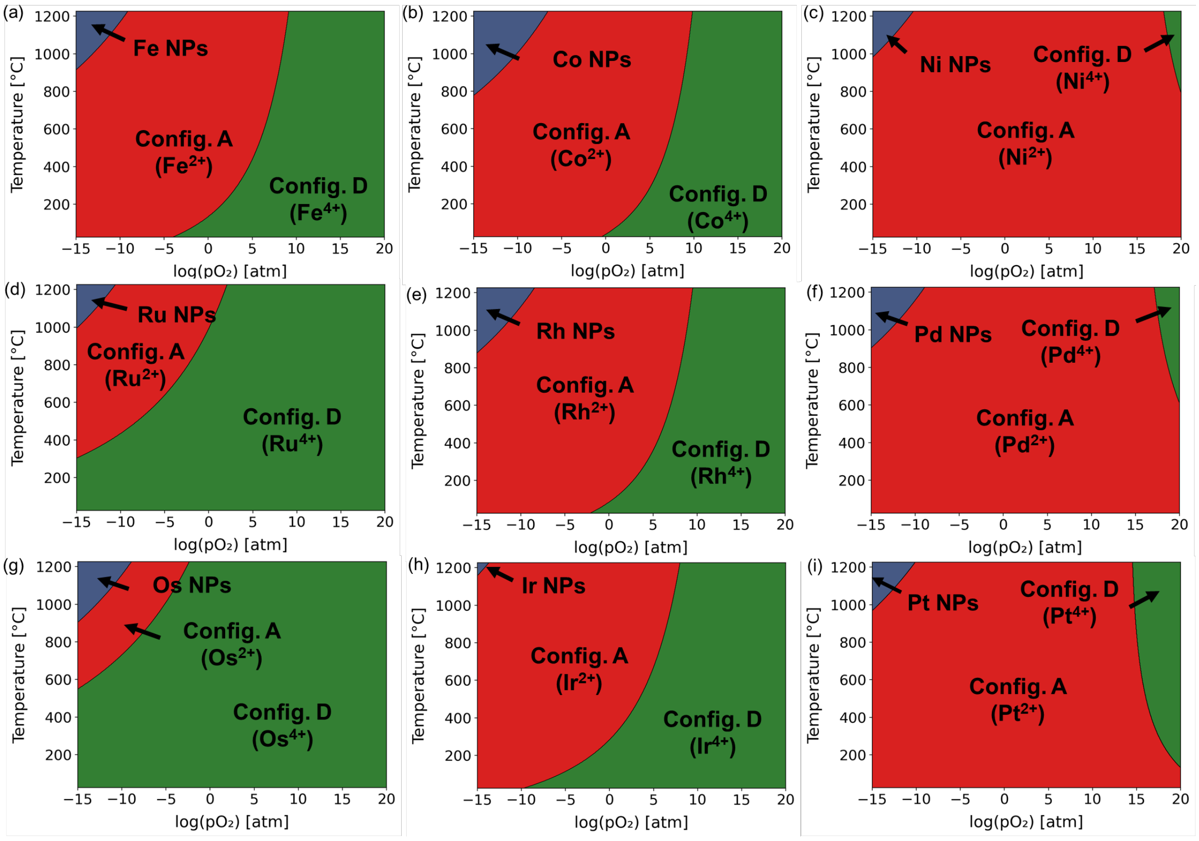

Figure S9. Stability diagrams of homonuclear DMSCs in configuration A vs large nanoparticles (NPs) and configuration D.

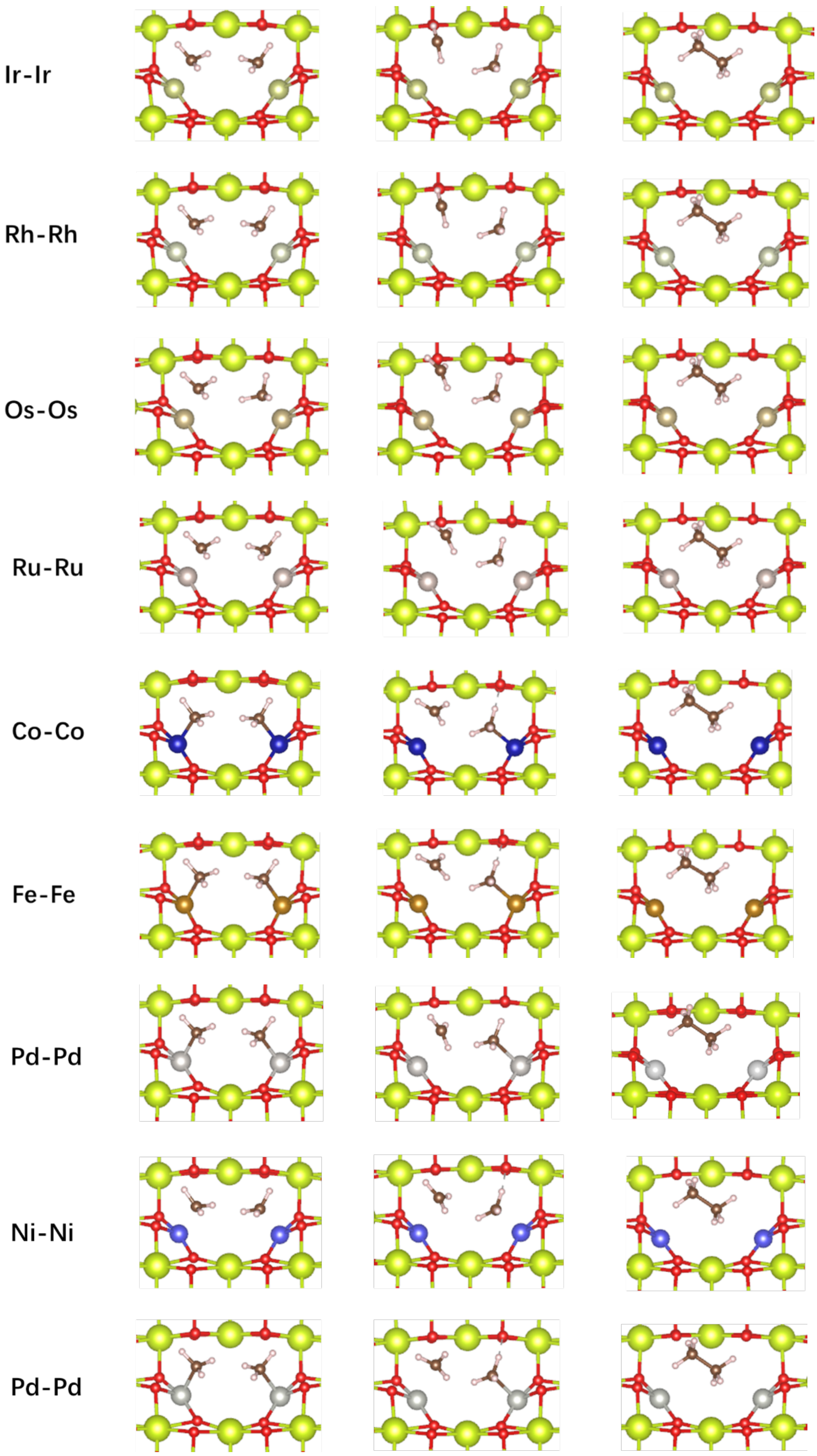

Figure S10. Structures of CH_3 co-adsorption, transition state of coupling, and ethane adsorption for homonuclear DMSCs.

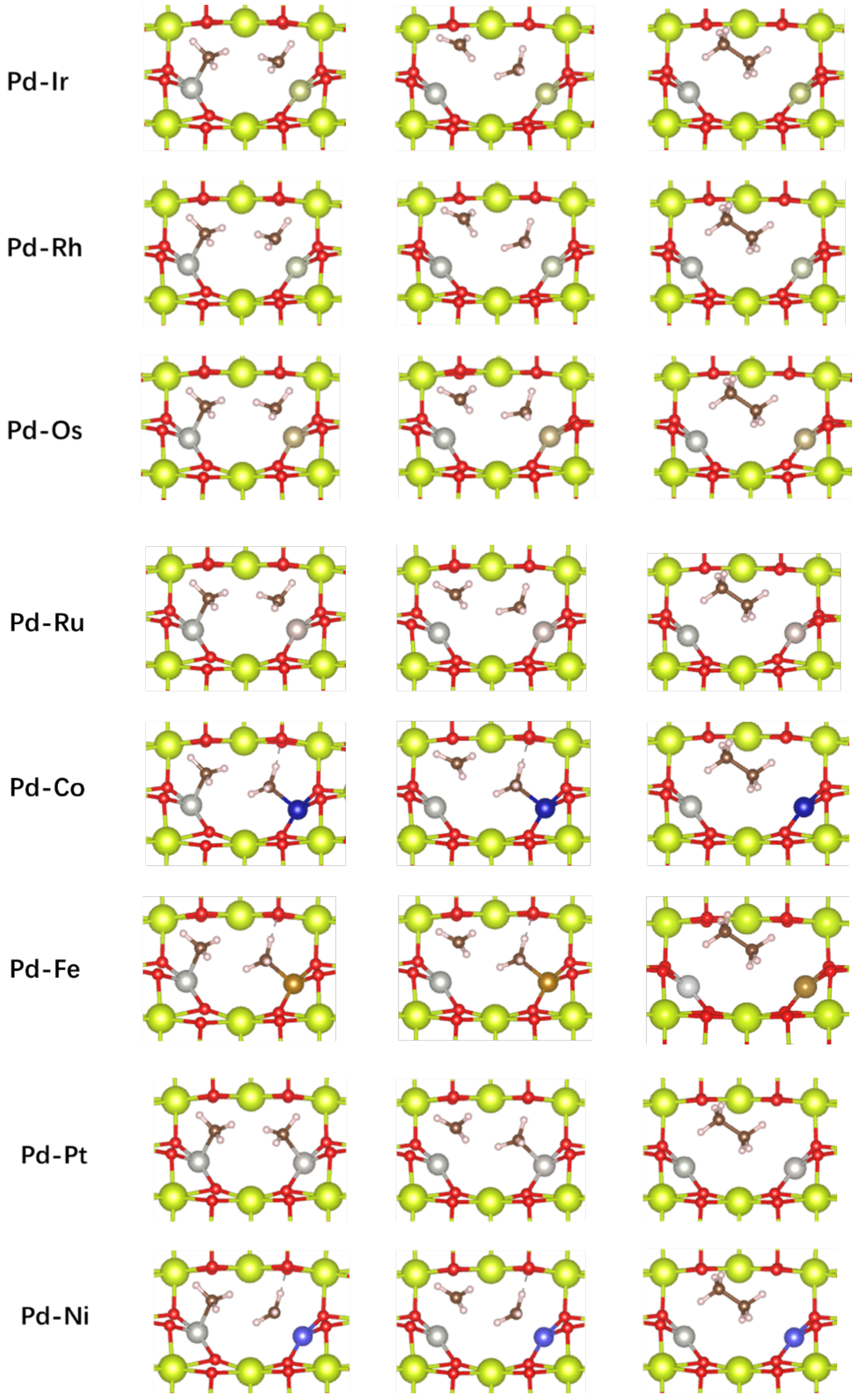

Figure S11. Structures of CH_3 co-adsorption, transition state of coupling, and ethane adsorption for Pd-based heteronuclear DMSCs. Pd metal sites are consistently posited on the left in all figures. Other heteronuclear catalysts exhibit similar structures across different adsorption and transition states when compared to Pd-based heteronuclear DMSCs.

Table S1. Substitution strategies (see Figure S1 for the codes), optimized configurations, and energies for possible Pd-Ni heteronuclear DMSCs. (Ce, green; O, red; Pd, grey; Ni, Blue)

Substitution Strategy	Configuration	Top View of Optimized Structure	Energy (eV)
Pd+uc+Ni+df	1	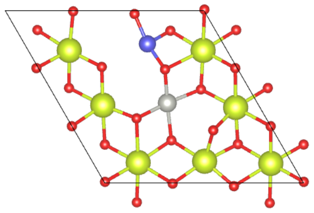	-639.53
Pd+uc+Ni+uf	2	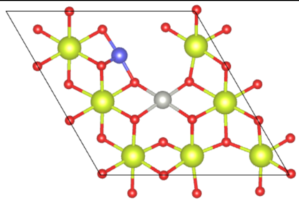	-639.64
Pd+uf+Ni+df	3	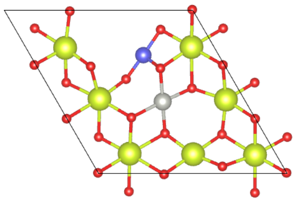	-639.73
Pd+df+Ni+uf	4	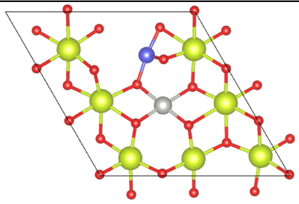	-639.74
Pd+df+Ni+df	5	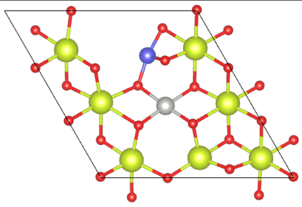	-639.76
Pd+um+Ni+dc	6	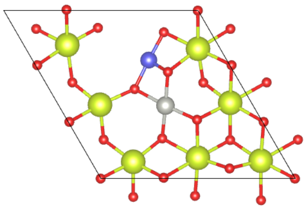	-640.06
Pd+um+Ni+um	7	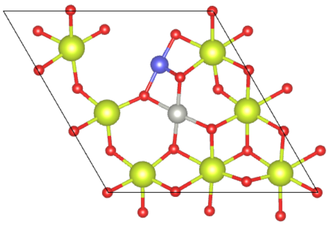	-640.06

Pd+df+Ni+dc	8	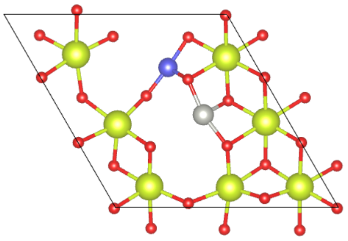	-640.33
Pd+uf+Ni+uf	9	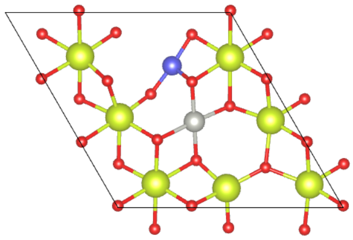	-640.33
Pd+dc+Ni+um	10	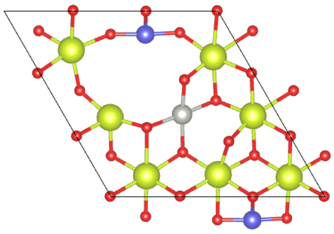	-640.35
Pd+um+Ni+df	11	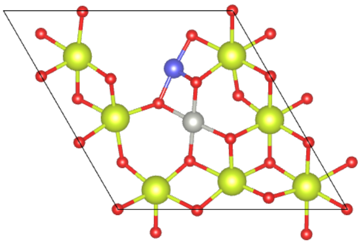	-640.41
Pd+uc+Ni+dm	12	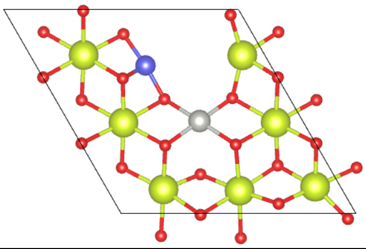	-640.44
Pd+dm+Ni+df	13	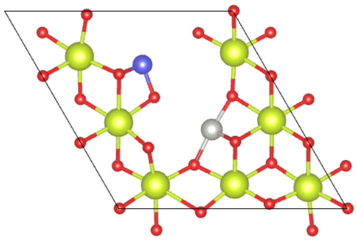	-640.52
Pd+um+Ni+uf	14	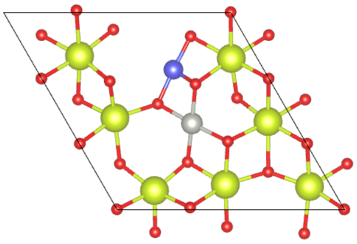	-640.55

Pd+uf+Ni+dc	15	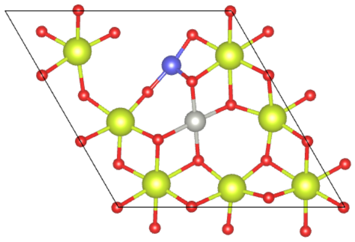	-640.62
Pd+uf+Ni+um	16	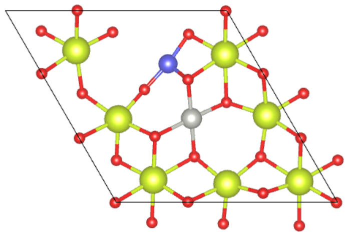	-640.62
Pd+df+Ni+um	17	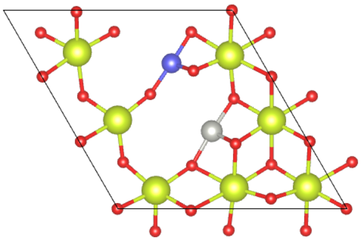	-640.74
Pd+uc+Ni+um	18	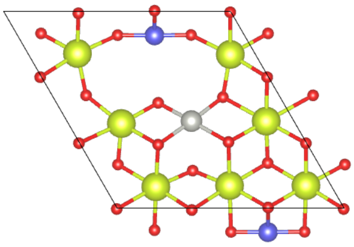	-640.81
Pd+dm+Ni+um	19	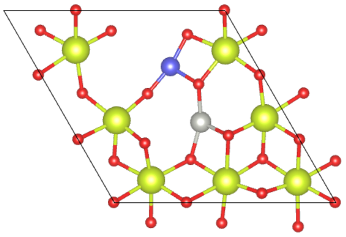	-640.94
Pd+dm+Ni+dc	20	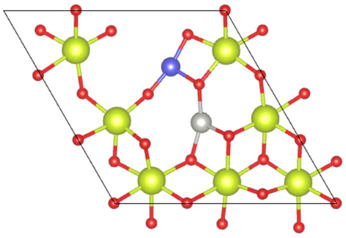	-640.94
Pd+uc+Ni+uf	21	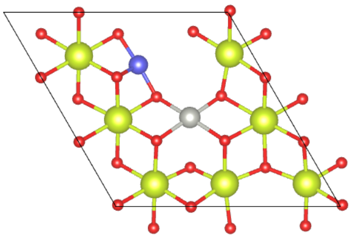	-640.95

Pd+uf+Ni+dm	22	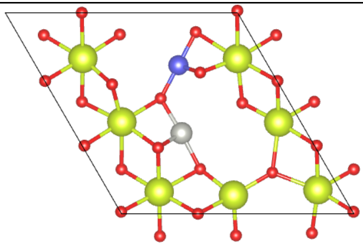	-641.10
Pd+um+Ni+dm	23	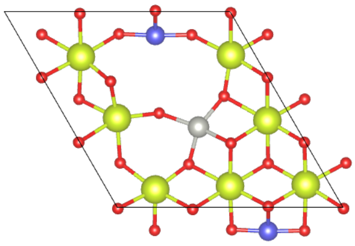	-641.19
Pd+df+Ni+uc	24	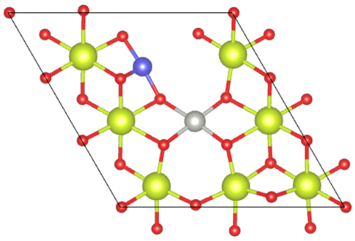	-641.24
Pd+df+Ni+dm	25	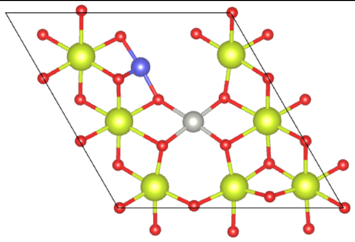	-641.24
Pd+dm+Ni+uc	26	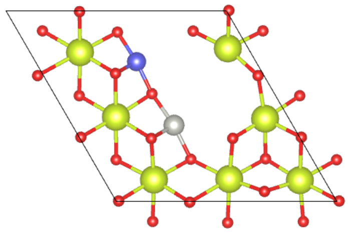	-641.42
Pd+dc+Ni+dm	27	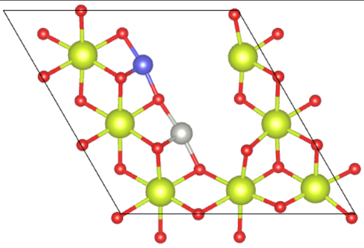	-641.43
Pd+dm+Ni+dm	28	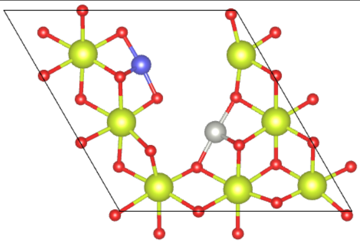	-641.61

Pd+uc+Ni+df	29	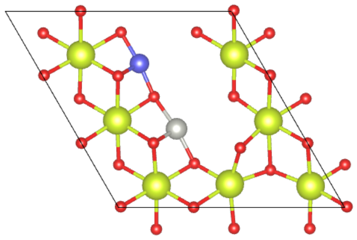	-641.83
Pd+uf+Ni+uc	30	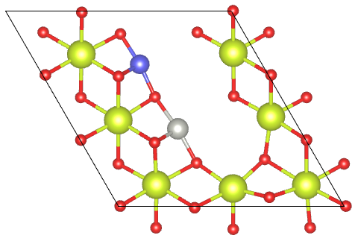	-641.88
Pd+dm+Ni+uf	31	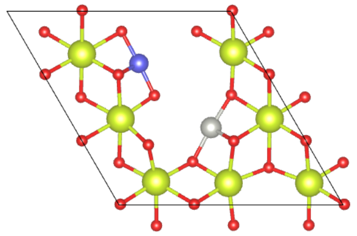	-641.99
d+um+Ni+uc	32	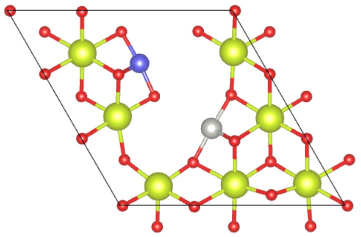	-642.10
Pd+uc+Ni+dc	33	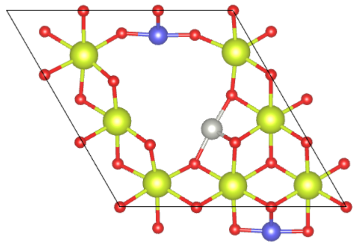	-642.55

Table S2. Two CH_3 co-adsorption energies, reaction energies, and activation energies on the DMSCs in configuration A.

	2 CH_3 co-adsorption energy (eV)	Energy difference (eV)	Activation energy (eV)
Homonuclear			
Ir-Ir	-5.08	-0.79	1.42
Rh-Rh	-4.98	-0.89	1.41
Os-Os	-4.66	-1.33	1.03
Ru-Ru	-4.2	-1.61	0.92
Co-Co	-4	-2.03	0.87
Fe-Fe	-3.02	-2.91	0.45
Pt-Pt	-2.95	-2.97	0.4
Ni-Ni	-2.76	-3.14	0.25
Pd-Pd	-2.14	-3.76	0.12
Pd based Heteronuclear			
Pd+Ni	-2.42	-3.47	0.21
Pd+Pt	-2.5	-3.39	0.1
Pd+Fe	-2.78	-3.38	0.19
Pd+Co	-3.23	-2.65	0.19
Pd+Ru	-3.12	-2.72	0.15
Pd+Os	-3.32	-2.52	0.05
Pd+Rh	-3.57	-2.31	0.06
Pd+Ir	-3.63	-2.25	0.03
Ni based Heteronuclear			
Ni+Pt	-2.56	-3.24	0.17
Ni+Fe	-2.77	-3.08	0.1
Ni+Co	-3.3	-2.75	0.25
Ni+Ru	-3.37	-2.47	0.6
Ni+Os	-3.54	-2.27	0.3
Ni+Rh	-3.67	-2.21	0.25
Ni+Ir	-3.73	-2.15	0.3
Pt based Heteronuclear			
Pt+Fe	-2.88	-2.85	0.36
Pt+Co	-3.65	-2.24	0.61
Pt+Ru	-3.53	-2.31	0.52
Pt+Os	-3.91	-2.13	0.45
Pt+Rh	-3.98	-1.91	0.43

Pt+Ir	-4.03	-1.86	0.43

Fe based Heteronuclear			
Fe+Co	-3.25	-2.59	0.54
Fe+Ru	-3.48	-2.47	0.26
Fe+Os	-3.5	-2.29	0.27
Fe+Rh	-3.71	-2.15	0.55
Fe+Ir	-3.88	-1.98	0.53

Co based Heteronuclear			
Co+Ru	-4.22	-1.67	1.17
Co+Os	-4.58	-1.52	1.2
Co+Rh	-4.39	-1.49	0.95
Co+Ir	-4.59	-1.28	1.05

Ru based Heteronuclear			
Ru+Os	-4.39	-1.44	1.02
Ru+Rh	-4.5	-1.35	1.25
Ru+Ir	-4.68	-1.19	1.1

Os based Heteronuclear			
Os+Rh	-4.76	-1.1	1.34
Os+Ir	-4.78	-1.23	1.26

Rh based Heteronuclear			
Rh+Ir	-4.71	-0.96	1.4

Coordinates of optimized structures of the initial, final, and transition states for the methyl coupling reaction on Ni–Ir DMSC configuration A and frequency information for the transition state

Initial State
1.0

11.4785003662	0.0000000000	0.0000000000
-5.7392501831	9.9406729145	0.0000000000
0.0000000000	0.0000000000	20.8101005554

O	Ce	Ni	Ir	C	H
52	25	1	1	2	6

Cartesian

0.009986300	0.007356100	3.127133813
-0.102736199	2.171711183	6.263381307
1.903307490	1.104508185	20.740179170
-0.002582664	2.207724106	1.573451639
-5.682467499	9.926658269	7.999914520
1.948346913	1.136703639	4.792954279
-1.912490445	9.939579733	3.111109981
3.828196841	2.335856824	6.415922454
5.755434783	1.116735228	20.768896417
3.816199800	2.194005864	1.555346917
3.858155439	0.041344640	7.971636368
5.685365101	1.068981640	4.781446666
1.898084521	9.890969597	3.136914508
7.669781900	2.193848108	6.273512720
9.558319230	1.110074968	20.776179920
7.665399865	2.207326383	1.632760499
7.636682485	0.202597391	8.083305177
9.553396613	1.037308780	4.746139908
-1.853698252	3.332232772	3.203384230
-1.959259669	5.458726682	6.265267923
-0.002525279	4.409483707	20.751415761
-1.909907782	5.514091434	1.574700235
9.461979354	3.261588068	8.024411378
0.220531535	4.530458308	4.930120312
1.914269634	3.309249891	3.088010719
3.842428301	4.414851554	20.759324415
1.917196522	5.521347908	1.533288178
1.406841373	3.064359764	8.205186644
3.685383646	4.461407160	5.006878974
5.686907951	3.324956153	3.132960491

5.598900178	5.559417878	6.514389293
7.650764657	4.402724054	20.779301954
5.752393126	5.529499072	1.586145829
5.800965742	3.295668074	7.850600839
7.659190748	4.389993674	4.794473123
-3.811034408	6.632317714	3.130879756
-3.751046107	8.858446967	6.342017593
-1.920008982	7.727084142	20.746421996
-3.823316215	8.841930150	1.563254796
-3.801323390	6.627898180	7.952119630
-1.885363541	7.725277578	4.766302573
0.002869759	6.629434559	3.081351437
-0.010507513	8.754768926	6.358678359
1.922476277	7.741498139	20.756826292
-0.014635122	8.837556239	1.554098321
-0.422004434	6.310976282	8.186833381
1.920610882	7.628584048	4.905487942
3.838812453	6.636691625	3.099664387
3.953734181	8.708640816	6.423175568
5.739479723	7.728872930	20.757658586
3.834622242	8.842228775	1.577613729
5.813092860	7.809482176	4.806456111
-0.000688700	0.004572700	0.766436016
1.887785681	1.181822778	7.228653897
0.020463497	2.210679103	3.978386455
3.824980534	0.000397600	0.740839562
5.711711536	1.056194675	7.154993390
3.814087933	2.216698711	3.948182615
7.655987605	0.001292300	0.773511368
9.551224580	1.076872691	7.146026073
7.641685525	2.196094018	3.976844975
9.566756352	3.310641698	0.790991869
-0.164006789	4.316858946	7.336113481
-1.911029824	5.532007760	3.962401695
1.911399875	3.300701153	0.727104926
1.930417461	5.518287000	3.857856381
5.742176900	3.313922131	0.774135666
7.675574613	4.404567057	7.105832843
5.733554452	5.537125864	3.981923705
-3.821881281	6.623271267	0.769973730
-1.854663207	7.648065786	7.226449124
-3.796888962	8.837661706	3.942781076
-0.008895946	6.629335610	0.727937298
-0.045476224	8.821733845	3.899818080

3.843575658	6.635597851	0.737509998
5.936807567	7.830342093	7.102715772
3.871034556	8.823775635	3.943488092
4.578490575	4.011276214	6.622900098
1.955012415	8.680780390	6.611109058
3.843731307	4.564462177	8.362598757
2.277472228	7.649279247	8.353718886
3.658308397	3.664870412	8.943299388
2.931472182	5.003758777	7.963508786
4.534757620	5.267136113	8.832849251
2.202439776	6.598978938	8.067669584
1.488445810	7.902369420	9.065681968
3.264132998	7.885145739	8.781429974

Final State

1.0

11.4785003662	0.0000000000	0.0000000000
-5.7392501831	9.9406729145	0.0000000000
0.0000000000	0.0000000000	20.8101005554

O	Ce	Ni	Ir	C	H
52	25	1	1	2	6

Cartesian

0.009986300	0.007356100	3.127133813
-0.091626957	2.189623065	6.274020035
1.903307490	1.104508185	20.740179170
-0.002582664	2.207724106	1.573451639
0.051639403	0.002747364	7.962447643
1.926698473	1.123685966	4.784245270
-1.912490445	9.939579733	3.111109981
3.817128478	2.328140859	6.326489292
5.755434783	1.116735228	20.768896417
3.816199800	2.194005864	1.555346917
3.852658984	0.064641237	7.932935313
5.691307091	1.093826855	4.764748689
1.898084521	9.890969597	3.136914508
7.665006722	2.211641784	6.301849791
9.558319230	1.110074968	20.776179920
7.665399865	2.207326383	1.632760499
7.656795486	0.022696361	8.001510887
9.569053674	1.066159661	4.750904822
-1.864788540	3.340380973	3.207792535
-1.992104113	5.455618965	6.270899862
-0.002525279	4.409483707	20.751415761
-1.909907782	5.514091434	1.574700235

9.475028224	3.269848254	8.025531440
0.193672142	4.530319957	4.918902327
1.914269634	3.309249891	3.088010719
3.842428301	4.414851554	20.759324415
1.917196522	5.521347908	1.533288178
1.518094914	3.158720581	8.113344667
3.741067203	4.494332660	4.878840888
5.686907951	3.324956153	3.132960491
5.625190511	5.564396742	6.406305798
7.650764657	4.402724054	20.779301954
5.752393126	5.529499072	1.586145829
5.728388098	3.330962726	7.842636368
7.599768191	4.425964084	4.755172965
-3.811034408	6.632317714	3.130879756
-3.776501322	8.841966293	6.321829190
-1.920008982	7.727084142	20.746421996
-3.823316215	8.841930150	1.563254796
-3.811524419	6.634928314	7.971135255
-1.878217194	7.724435028	4.769673302
0.002869759	6.629434559	3.081351437
0.003461568	8.799943094	6.358825964
1.922476277	7.741498139	20.756826292
-0.014635122	8.837556239	1.554098321
-0.334035317	6.375629226	8.097392157
1.911369430	7.564011686	4.880166852
3.838812453	6.636691625	3.099664387
3.930601644	8.676840789	6.429070467
5.739479723	7.728872930	20.757658586
3.834622242	8.842228775	1.577613729
5.788677158	7.739946947	4.760163939
-0.000688700	0.004572700	0.766436016
1.893799723	1.213846477	7.208319129
0.016368899	2.209749602	3.975996866
3.824980534	0.000397600	0.740839562
5.668564660	1.135840351	7.130308615
3.810536140	2.217946982	3.943195362
7.655987605	0.001292300	0.773511368
9.571247343	1.099672857	7.140126832
7.641268094	2.188520700	3.989265816
9.566756352	3.310641698	0.790991869
-0.133777165	4.347356338	7.323261298
-1.895563176	5.532277352	3.973352688
1.911399875	3.300701153	0.727104926
1.925301907	5.507854671	3.846954693

5.742176900	3.313922131	0.774135666
7.629696096	4.405894280	7.126643916
5.734820512	5.536647708	3.958527993
-3.821881281	6.623271267	0.769973730
-1.851167944	7.656755542	7.211141611
-3.803062930	8.847027516	3.947999349
-0.008895946	6.629335610	0.727937298
-0.022404578	8.834011250	3.921632620
3.843575658	6.635597851	0.737509998
5.924080600	7.801581644	7.112599729
3.838385529	8.831214602	3.960722843
4.660307618	3.973127439	6.440090611
1.964456725	8.728540273	6.486482634
3.937588750	5.522240821	9.997330993
3.726653752	6.846440856	9.271572420
3.126459645	4.819848623	9.758603376
4.874052167	5.036524003	9.688576559
3.964510573	5.665154892	11.085679534
3.659862151	6.683169909	8.181541305
2.784077259	7.342441282	9.540580780
4.523581330	7.578719568	9.508726616

Transition State (the one and only imaginary frequency is 127.77 cm^{-1})

1.0

11.4785003662	0.0000000000	0.0000000000
-5.7392501831	9.9406729145	0.0000000000
0.0000000000	0.0000000000	20.8101005554

O	Ce	Ni	Ir	C	H
52	25	1	1	2	6

Cartesian

0.009986300	0.007356100	3.127133813
-0.115816625	2.177280928	6.256590854
1.903307490	1.104508185	20.740179170
-0.002582749	2.207724106	1.573451639
-5.691878468	9.927093764	7.984636156
1.931486650	1.125505565	4.787580338
-1.912490103	9.939579733	3.111109981
3.807614897	2.340493217	6.369271813
5.755434783	1.116735228	20.768896417
3.816199715	2.194006012	1.555346917
3.829611046	0.050856898	7.948337095
5.690686419	1.070781982	4.767448993
1.898084863	9.890969005	3.136914508
7.684753207	2.215096267	6.241319933

9.558319230	1.110074968	20.776179920
7.665399865	2.207326383	1.632760499
7.628515493	0.199119411	8.066557584
9.553779407	1.053247899	4.725222472
-1.858287111	3.347198989	3.201855463
-1.988900734	5.460168259	6.251662830
-0.002525279	4.409484003	20.751415761
-1.909907782	5.514091434	1.574700235
9.455617239	3.272375310	8.018716800
0.208041292	4.533573727	4.928179739
1.914269634	3.309249891	3.088010719
3.842428301	4.414851554	20.759324415
1.917196522	5.521347908	1.533288178
1.413652305	3.106197507	8.155572118
3.724586185	4.500031424	4.925822710
5.686907951	3.324956153	3.132960491
5.622562607	5.574160126	6.410785425
7.650764486	4.402724350	20.779303194
5.752393126	5.529499072	1.586145829
5.813855541	3.297452715	7.825169355
7.598522314	4.440120340	4.740943962
-3.811034540	6.632317714	3.130879756
-3.792726888	8.847535890	6.315870411
-1.920008982	7.727084142	20.746421996
-3.823315958	8.841930150	1.563254796
-3.829556256	6.648796610	7.957165490
-1.889621656	7.734110129	4.764620929
0.002869759	6.629434559	3.081351437
-0.000981103	8.797148816	6.357545273
1.922476277	7.741498139	20.756826292
-0.014635122	8.837555647	1.554098321
-0.429115718	6.355329824	8.156909246
1.911044106	7.611453391	4.877748424
3.838812453	6.636691625	3.099664387
3.966302762	8.741529284	6.356987102
5.739479723	7.728872930	20.757658586
3.834622242	8.842228775	1.577613729
5.795994035	7.734919498	4.751470435
-0.000688700	0.004572700	0.766436016
1.881312303	1.197475566	7.218928088
0.014955485	2.211404632	3.977998837
3.824980534	0.000397600	0.740839639
5.693174838	1.108779518	7.149908458
3.809903623	2.221283407	3.967685398

7.655987606	0.001292300	0.773511445
9.546891206	1.090426660	7.145479686
7.641019911	2.194119626	3.974389645
9.566756352	3.310641698	0.790991869
-0.174174781	4.332140971	7.324143827
-1.901233936	5.535539714	3.966371837
1.911399875	3.300701153	0.727104926
1.926004894	5.516995920	3.861791482
5.742176900	3.313922131	0.774135744
7.635531740	4.407858452	7.102971290
5.731937069	5.534111172	3.963561449
-3.821881180	6.623271267	0.769973730
-1.871490245	7.671588444	7.216561445
-3.803631220	8.848249272	3.943086209
-0.008895946	6.629335610	0.727937298
-0.046071112	8.823994864	3.898081240
3.843575658	6.635597851	0.737509998
5.916025502	7.813374376	7.098099082
3.853117120	8.831578403	3.948245874
4.610209812	4.007709894	6.538610167
1.958820858	8.724888633	6.565069924
3.845817176	4.659793822	8.952308878
2.457606489	7.560893304	8.175867813
4.090797082	3.696909220	9.386984072
2.795667474	4.863809627	8.781998688
4.533931825	5.477178637	9.167231768
2.878834046	6.637674599	7.761690497
1.519388512	7.365253520	8.708127317
3.171964091	8.083535955	8.829427666

References

(1) Nosé, S. A Unified Formulation of the Constant Temperature Molecular Dynamics Methods. *J. Chem. Phys.* **1984**, *81* (1), 511–519. https://doi.org/10.1063/1.447334.

(2) Hoover, W. G. Canonical Dynamics: Equilibrium Phase-Space Distributions. *Phys. Rev. A* **1985**, *31* (3), 1695–1697. https://doi.org/10.1103/PhysRevA.31.1695.

(3) Mayernick, A. D.; Janik, M. J. Ab Initio Thermodynamic Evaluation of Pd Atom Interaction with CeO2 Surfaces. *J. Chem. Phys.* **2009**, *131* (8), 084701. https://doi.org/10.1063/1.3207283.

(4) Malcolm W. Chase, J. *NIST-JANAF Thermochemical Tables*; Fourth edition. Washington, DC : American Chemical Society ; New York : American Institute of Physics for the National Institute of Standards and Technology, 1998., 1998.

(5) Reuter, K.; Scheffler, M. Composition and Structure of the RuO_2(110) Surface in an O_2 and CO Environment: Implications for the Catalytic Formation of CO_2. *Phys. Rev. B* **2003**, *68* (4), 045407. https://doi.org/10.1103/PhysRevB.68.045407.

(6) Su, Y.-Q.; Filot, I. A. W.; Liu, J.-X.; Hensen, E. J. M. Stable Pd-Doped Ceria Structures for CH4 Activation and CO Oxidation. *ACS Catal.* **2018**, *8* (1), 75–80. https://doi.org/10.1021/acscatal.7b03295.
