# Supplementary material for: Breaking the Brønsted–Evans–Polanyi Relation with Dual-Metal Sites
Source: J Phys Chem Lett. 2025 Oct 23;16(43):11302–7. doi: 10.1021/acs.jpclett.5c02446 (PMC12581165; doi:10.1021/acs.jpclett.5c02446)
Supplement: Supplementary file 3 [file jz5c02446_si_003.pdf]

jz-2025-024469.R1

Name: Peer Review Information for "Breaking the Brønsted−Evans−Polanyi Relation with Dual-Metal Sites"

First Round of Reviewer Comments

Reviewer: 1

Comments to the Author

The authors present a dual-metal site catalyst (DMSC) on ceria that breaks the BEP relation for C–C coupling of methyl intermediates. They found that many heteronuclear DMSCs break the BEP linear scaling due to a mixed low-affinity/high-affinity co-adsorption of the two methyl groups. The full manuscript is well organized and analyzed with strict logic, and the results are reasonable and convincing. However, some questions need to be clarified before the manuscript can be considered for publication.

1) The manuscript notes that many heteronuclear DMSCs break the BEP linear scaling relationship through a mechanism described as “mixed low-affinity/high-affinity co-adsorption.” Could the authors provide a more in-depth explanation of the specific details of this mechanism? For instance, during the reaction process, how do the low-affinity and high-affinity sites coordinate such that the activation energy is primarily governed by the low-affinity site, while the overall reaction energy is largely determined by the high-affinity site?

2) Is this decoupling mechanism universal? Could it be extended to other types of C-C coupling reactions or other catalytic reactions beyond methyl coupling?

3) The manuscript mentions that a stable DMSC structure on CeO_2(111) was explored and identified through density functional theory (DFT) calculations, and its high stability was confirmed by 10 ps of AIMD simulations at 1000 K. Given that practical catalytic reactions, particularly oxidative coupling of methane (OCM), typically occur at high temperatures, is a 10 ps simulation duration sufficient to adequately demonstrate the long-

term thermal stability of the DMSC and effectively rule out potential issues such as sintering and aggregation under such conditions?

4) Since this is a theoretical computational study, are there any plans or existing experimental work aimed at validating these computational results, especially regarding the structural stability and catalytic performance of the DMSC under actual reaction conditions?

5) The manuscript emphasizes the inherent limitations that linear scaling relationships (LSRs) and BEP relations impose on catalytic activity. This study overcomes these constraints using DMSCs, thereby proposing new principles for catalyst design. Compared to previous approaches aimed at breaking the limitations of "volcano plots" or LSRs—such as modulating catalyst elements or designing alloy catalysts—what are the unique advantages and potential limitations of DMSCs?

6) It is noted that DMSCs, similar to single-atom catalysts (SACs), are prone to sintering and aggregation at high temperatures, aside from the stabilizing effect of the CeO_2(111) support, what other strategies could further enhance the stability of DMSCs for practical applications?

Reviewer: 2

Comments to the Author

The authors report a DFT study demonstrating that dual-metal sites (DMSCs) doped in CeO2(111) appear to break the BEP scaling relationship between CH3 adsorption and C-C coupling during the methane coupling reaction. The data support the hypothesis that one can break the BEP relationship by pairing a metal with high methyl affinity with one that has a low affinity. Overall, this is an intriguing concept and the results look promising; the paper will be of broad interest to the readership of this journal. However, there are some areas that need improvement before publication, as described in the points below. For this reason, I recommend that the paper be re-evaluated following major revisions.

1. Major: One aspect of the work that I am concerned with is the claim of high DMSC site stability. In the introduction the authors highlight the importance of site stability against sintering and imply that the DMSC sites are unusually stable (e.g., compared to other

single-atom sites reported in the literature). The data supporting this claim are two 10 ps AIMD simulations, which indeed do show little egress of the metal atoms away from the ceria lattice. However, confirmation of stability against sintering needs to go beyond AIMD simulations, as I expect that almost all metal-doped structures would show similar stability over such a small timescale. AIMD cannot reach timescales that are relevant for describing the long-term stability of single/dual metal sites under methane coupling conditions. The authors instead should conduct an ab initio thermodynamics study to assess the expected long-term stability of the various DMSC sites based on formation energies. For example, see relevant examples from Janik (J. Chem. Phys. 2009 131, 084701) and from Hensen (ACS Catal. 2018, 8, 1, 75–80). Furthermore, it appears that the AIMD simulations were only conducted for Pd-Ni and Pd-Pd DMSCs, and it is assumed that the other DMSC combinations would also be stable. There is no obvious reason why the stability of these two DMSC combinations would apply also to all the other combinations. AIMD simulations would be expensive to repeat for all the studied DMSC combinations, but a thermodynamic analysis based on formation energies would be feasible.

2. Major: In addition, the stability analysis should assess the amount of oxygen adsorbed on each of the DMSC sites. The current site model assumes an oxygen stoichiometry that forces a formal 2+ oxidation state on each metal atom, but this likely is not he most stable oxidation state for all of the DMSCs. My concern with this approach is that the arbitrary choice of site stoichiometry may be forcing some of the metal centers into unphysical (unstable) oxidation states, which in turn may be responsible for observed breaking of the BEP relationships. A thermodynamic analysis of the oxidation state stability would greatly improve the understanding of the reactivity of the site and will help identify the most promising DMSC combinations for experimental efforts to target.

3. Minor: Coordinates of the relaxed and transition state structures (as well as the associated vibrational frequencies for TS saddle point confirmation) need to be reported in the SI.

4. Minor: The AIMD settings need to be discussed in greater detail (missing information about the timestep, thermostat, equilibration time, etc.).

Author's Response to Peer Review Comments:

Dear editor,

We thank the two reviewers for their positive comments and have addressed their specific comments in our response letter attached. The detailed changes to the manuscript have been described in the response letter as well as highlighted in a marked copy as Supporting Information for Review Only.

We also addressed the editorial comments regarding formatting.

Thanks again.

De-en

Referee: 1

Comments to the Author

The authors present a dual-metal site catalyst (DMSC) on ceria that breaks the BEP relation for C–C coupling of methyl intermediates. They found that many heteronuclear DMSCs break the BEP linear scaling due to a mixed low-affinity/high-affinity co-adsorption of the two methyl groups. The full manuscript is well organized and analyzed with strict logic, and the results are reasonable and convincing. However, some questions need to be clarified before the manuscript can be considered for publication.

[Author reply:] We thank the reviewer for the positive feedback on our work and address the specific comments below.

Here are my detailed comments:

1) The manuscript notes that many heteronuclear DMSCs break the BEP linear scaling relationship through a mechanism described as “mixed low-affinity/high-affinity coadsorption.” Could the authors provide a more in-depth explanation of the specific details of this mechanism? For instance, during the reaction process, how do the low-affinity and highaffinity sites coordinate such that the activation energy is primarily governed by the lowaffinity site, while the overall reaction energy is largely determined by the high-affinity site?

[Author reply:] As shown in Figure 4, the Ni-Ir/CeO_2(111) DMSC is used as a specific example to explain the details of the mixed low-affinity/high-affinity co-adsorption in breaking the BEP scaling for the methyl-methyl coupling on. Specifically, in the rate-limiting step of the first methyl detachment/activation (Figure 4b), it takes place at the low-affinity site (Ni in this case), so the activation energy is primarily governed by the energy required to break the Ni-CH_3 bond at this low affinity site and the subsequent, second methyl detachment/activation from the highaffinity site (Ir in this case) and the following methyl-methyl coupling is greatly facilitated by the approaching first methyl group, leading to a lower activation energy (Figure 4d) than the first one (Figure 4b). On the other hand, the overall reaction energy is determined by the total energy of

dissociating both methyl groups from the two metal sites and the formation of the CH_3-CH_3 bond, which is the result of the combined effects of *both* low-affinity and highaffinity sites. Because the rate-limiting step is the metal-methyl bonding breaking at the lowaffinity site while the overall reaction energy is due to the metal-methyl bonding breakings at both low-affinity and high-affinity sites, the linear scaling is broken. We have added this detailed discussion in the revised manuscript:

(pp.7-8) "In the rate-limiting step of the first methyl detachment/activation (Figure 4b), it takes place at the LA site (Ni in this case), so the activation energy is primarily governed by the energy required to break the Ni-CH_3 bond at this LA site and the subsequent, second methyl detachment/activation from the HA site (Ir in this case) and the following methyl-methyl coupling is greatly facilitated by the approaching first methyl group, leading to a lower activation energy (Figure 4d) than the first one (Figure 4b). On the other hand, the overall reaction energy is determined by the total energy of dissociating both methyl groups from the two metal sites and the formation of the CH_3-CH_3 bond, which is the result of the combined effects of *both* LA and HA sites. Because the rate-limiting step is the metal-methyl bonding breaking at the LA site while the overall reaction energy is due to the metal-methyl bonding breakings at both LA and HA sites, the linear scaling is broken."

2) Is this decoupling mechanism universal? Could it be extended to other types of C-C coupling reactions or other catalytic reactions beyond methyl coupling?

[Author reply:] We thank the reviewer for this thoughtful question. While our study uses the methyl coupling as a specific example to illustrate the proposed decoupling strategy, the mechanism arises from the intrinsic difference in adsorption strengths between two sites. As a result, in principle, this decoupling mechanism could be applied to other C-C coupling reactions and even broader processes involving two active sites with distinct affinity. However, the applicability of this mechanism might depend on the electronic and structural properties of reactants and active sites, which needs further study to test the universality of the mechanism. We have added some discussion in the revised manuscript:

(p.8 middle) "Our study uses methyl coupling as an example to illustrate a decoupling strategy arising from intrinsic differences in adsorption strengths between two sites in the DMSCs, which in principle could extend to other C–C coupling reactions and broader processes involving dual active sites. However, its broader applicability may depend on the electronic and structural properties of the reactants and sites, requiring further study to test its universality."

3) The manuscript mentions that a stable DMSC structure on CeO_2(111) was explored and identified through density functional theory (DFT) calculations, and its high stability was confirmed by 10 ps of AIMD simulations at 1000 K. Given that practical catalytic reactions, particularly oxidative coupling of methane (OCM), typically occur at high temperatures, is a 10 ps simulation duration sufficient to adequately demonstrate the long-term thermal stability of the

DMSC and effectively rule out potential issues such as sintering and aggregation under such conditions?

[Author reply:] We agree that 10 ps of AIMD simulations cannot fully capture long-term thermal stability. To address this limitation, we performed ab initio thermodynamic analyses comparing the stability of the most DMSC configuration identified in this work (called config A) to two previously reported configurations (config B, ref. 31; config C, ref. 32). As shown in Figure S8, config A remain the most stable structure at 600 °C for a range of O_2 pressures (pO_2) than config B and config C. We further examined the stability of config A against sintering to large metal nanoparticles or oxidation to the 4+ state: stability diagrams in Figure S9 show that config A is stable in phase fields of certain temperature and pO_2 ranges and the areas of the phase fields are especially large for group 9 and group 10 elements. We have added the details of the ab initio thermodynamics analysis in the SI and the following discussion in the revised manuscript:

(p.4) "To further evaluate the stability of the DMSCs against sintering and oxidation, we performed ab initio thermodynamics analysis following two previous studies.[33,34] We compared config A that we found this work (Figure 2a) with DMSCs from the literature and a typical 4+ configuration where the two metal atoms simply replace two Ce^{4+} on the surface (Figure S7). As shown in Figure S8, config A remain the most stable structure at 600 °C for a

range of O_2 pressures (pO_2). We further examined the stability of config A against sintering to large metal nanoparticles or oxidation to the 4+ state: stability diagrams in Figure S9 show that config A is stable in phase fields of certain temperature and pO_2 ranges and the areas of the phase fields are especially large group 9 and group 10 elements."

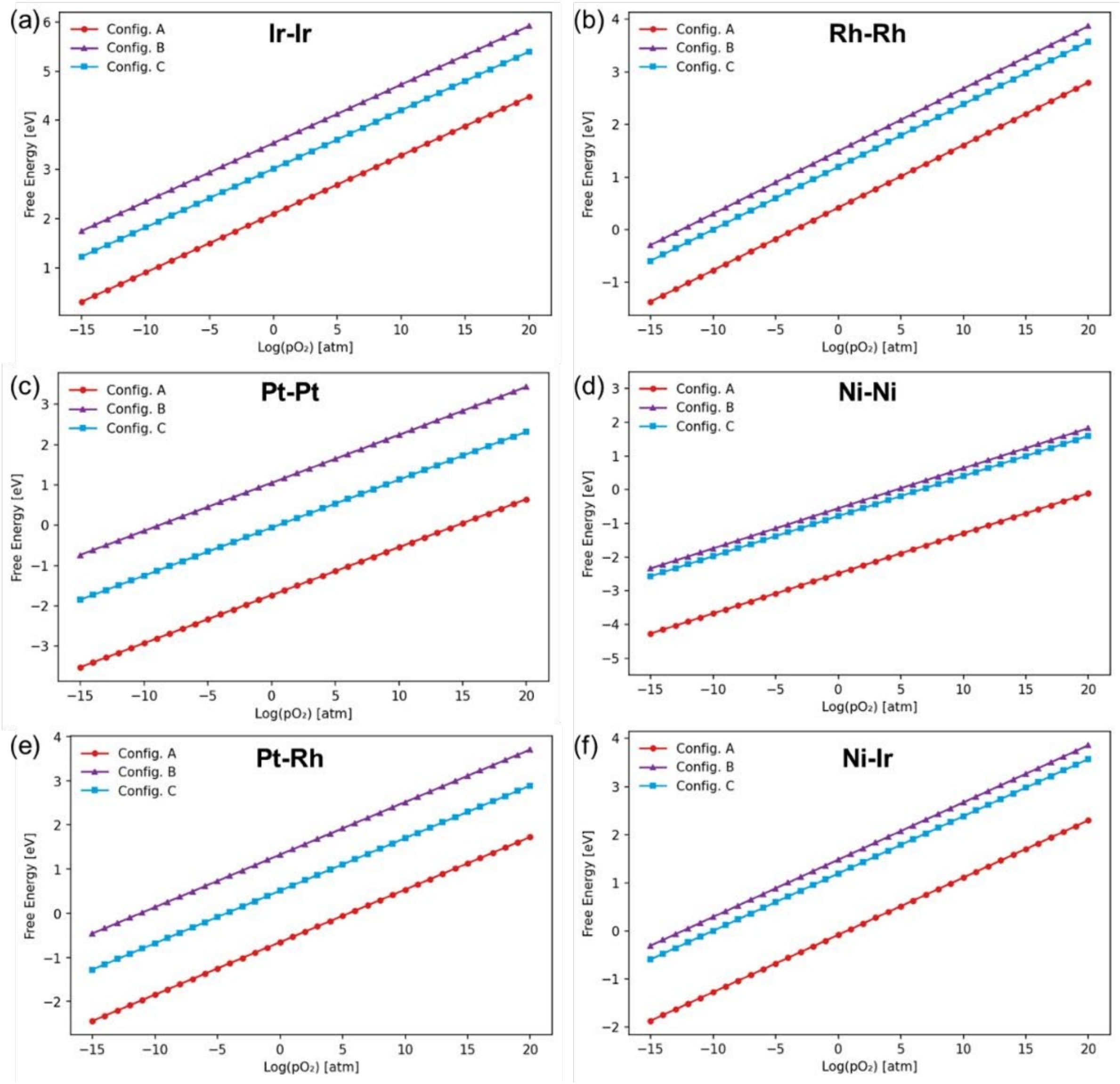

Figure S8. Relative stability of configurations A, B, and C for Ir-Ir, Rh-Rh, Pt-Pt, Ni-Ni, Pt-Rh, Ni-Ir DMSCs at 600 ºC.

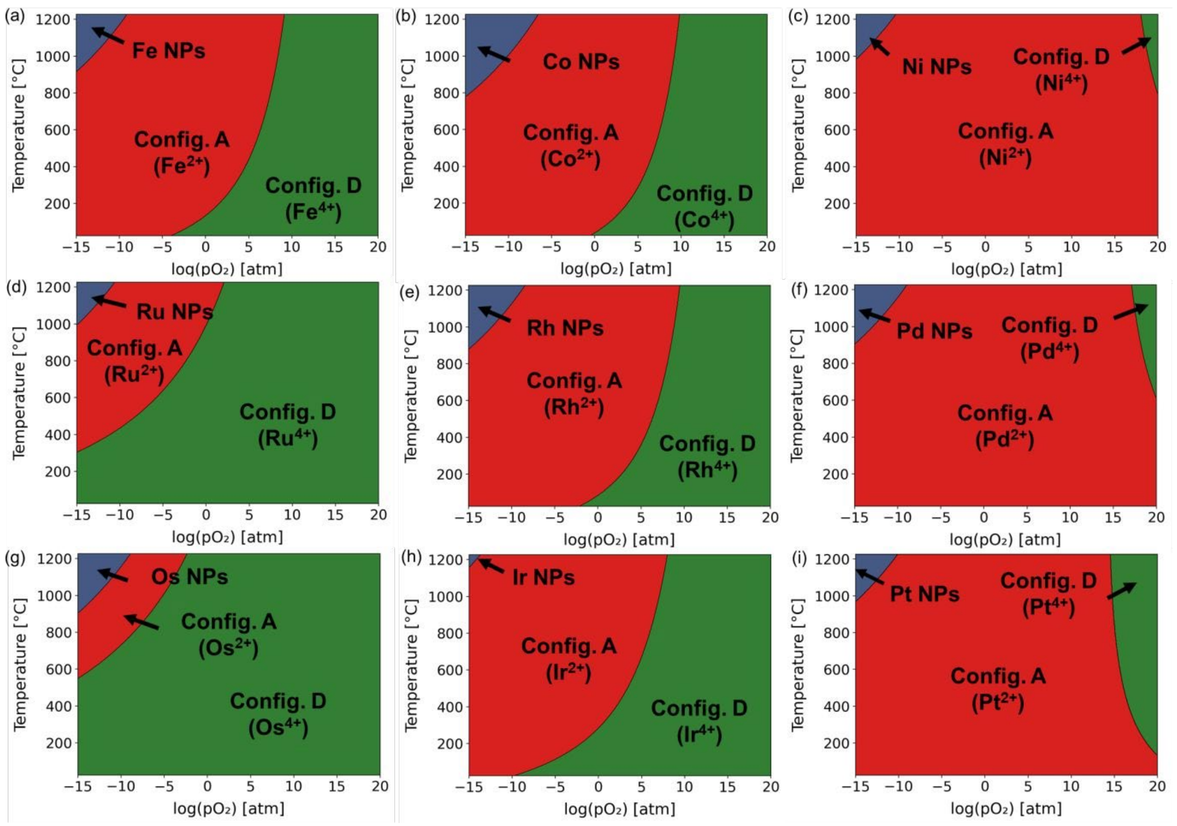

Figure S9. Stability diagrams of homonuclear DMSCs in Config. A vs large nanoparticles and Config. D.

4) Since this is a theoretical computational study, are there any plans or existing experimental work aimed at validating these computational results, especially regarding the structural stability and catalytic performance of the DMSC under actual reaction conditions?

[Author reply:] Yes, we're indeed exploring collaborations with experimental groups to test the predictions and stability of the proposed DMSC catalysts. Notably, we recently achieved a successful joint computational–experimental study on Pd_2 DMSC on CeO_2 for methane combustion (ref. 31).

5) The manuscript emphasizes the inherent limitations that linear scaling relationships (LSRs) and BEP relations impose on catalytic activity. This study overcomes these constraints using DMSCs, thereby proposing new principles for catalyst design. Compared to previous approaches aimed at breaking the limitations of "volcano plots" or LSRs—such as modulating catalyst elements or designing alloy catalysts—what are the unique advantages and potential limitations of DMSCs?

[Author reply:] We thank the reviewer for raising this important point. Compared with previous strategies for breaking volcano plot or LSR constraints, DMSCs offer a unique advantage: they

provide two adjacent atomic sites with intrinsically different affinities that can decouple activation energy from reaction energy. This decoupling mechanism allows us to go beyond the conventional linear correlations, enabling catalytic pathways that were previously inaccessible under LSR/BEP constraints. Additionally, DMSCs can combine the benefits of earth-abundant and noble metals, balancing activity, selectivity, and cost. At the same time, DMSCs also have potential limitations. They require precise positioning of the two metal sites on the support surface. Although our results demonstrate their intrinsic stability, realizing the specific configurations we identified may demand considerable synthetic ingenuity and extensive trial-and-error optimization.

6) It is noted that DMSCs, similar to single-atom catalysts (SACs), are prone to sintering and aggregation at high temperatures, aside from the stabilizing effect of the $CeO_2(111)$ support, what other strategies could further enhance the stability of DMSCs for practical applications?

[Author reply:] There are several strategies to enhance the stability of DMSCs. One promising approach is support doping, where dopants in the support interact strongly with metal sites and effectively 'anchor' them, thereby reducing the possibility of site migration and aggregation. For example, Ti doping led to exceptional stability rhodium catalyst on CeO_2 (Angewandte Chemie International Edition 2022, 61 (44), e202210991). Another approach is via surface functional groups. For example, hydroxyl-modified ceria improved sintering resistance of supported Pd sites (Journal of Materials Chemistry A 2023, 11, 21285–21292). We have added this discussion in the revised manuscript:

(p.8) "In addition, DMSCs like SACs are prone to sintering; recent studies show that support engineering (such as via Ti doping or steaming to create surface -OH groups) can greatly enhance the SAC stability on the ceria support. Such strategies can also be leveraged for stabilizing DMSCs on ceria."

Referee: 2

Comments to the Author

Comments:

The authors report a DFT study demonstrating that dual-metal sites (DMSCs) doped in CeO2(111) appear to break the BEP scaling relationship between CH3 adsorption and C-C coupling during the methane coupling reaction. The data support the hypothesis that one can break the BEP relationship by pairing a metal with high methyl affinity with one that has a low affinity. Overall, this is an intriguing concept and the results look promising; the paper will be of broad interest to the readership of this journal. However, there are some areas that need improvement before publication, as described in the points below. For this reason, I recommend that the paper be re-evaluated following major revisions.

[Author reply:] We thank the reviewer for the concise summary of our work and address the specific comments below.

1. Major: One aspect of the work that I am concerned with is the claim of high DMSC site stability. In the introduction the authors highlight the importance of site stability against sintering and imply that the DMSC sites are unusually stable (e.g., compared to other singleatom sites reported in the literature). The data supporting this claim are two 10 ps AIMD simulations, which indeed do show little egress of the metal atoms away from the ceria lattice. However, confirmation of stability against sintering needs to go beyond AIMD simulations, as I expect that almost all metal-doped structures would show similar stability over such a small timescale. AIMD cannot reach timescales that are relevant for describing the long-term stability of single/dual metal sites under methane coupling conditions. The authors instead should conduct an ab initio thermodynamics study to assess the expected long-term stability of the various DMSC sites based on formation energies. For example, see relevant examples from Janik (J. Chem. Phys. 2009 131, 084701) and from Hensen (ACS Catal. 2018, 8, 1, 75–80).

Furthermore, it appears that the AIMD simulations were only conducted for Pd-Ni and Pd-Pd DMSCs, and it is assumed that the other DMSC combinations would also be stable. There is no obvious reason why the stability of these two DMSC combinations would apply also to all the other combinations. AIMD simulations would be expensive to repeat for all the studied DMSC combinations, but a thermodynamic analysis based on formation energies would be feasible.

[Author reply:] Since this comment is highly related to the next one, we address them below together.

2. Major: In addition, the stability analysis should assess the amount of oxygen adsorbed on each of the DMSC sites. The current site model assumes an oxygen stoichiometry that forces a formal 2+ oxidation state on each metal atom, but this likely is not the most stable oxidation state for all of the DMSCs. My concern with this approach is that the arbitrary choice of site stoichiometry may be forcing some of the metal centers into unphysical (unstable) oxidation states, which in turn may be responsible for observed breaking of the BEP relationships. A thermodynamic analysis of the oxidation state stability would greatly improve the understanding of the reactivity of the site and will help identify the most promising DMSC combinations for experimental efforts to target.

[Author reply:] The reviewer raised two important points regarding the stability of DMSCs: stability against sintering to large metal nanoparticles (0 oxidation state); stability against oxidation to higher oxidation state (such as 4+). As the reviewer suggested, we performed ab initio thermodynamics analysis of several more stable configurations of the DMSCs on CeO_2: Config A is the one used in the text that shows the linear-scaling-breaking behavior; config B is from ref. 31; config C is from ref. 32 (Figure S7). We also compared with a typical 4+ configuration where M1 and M2 simply replace two Ce^{4+} on the surface (config D in Figure S7). As shown in Figure S8, config A remain the most stable structure at 600 °C for a range of O_2 pressures (pO_2) than config B and config C. We further examined the stability of config A against sintering to large metal nanoparticles: stability diagrams in Figure S9 show that config A is stable in phase fields of certain temperature and pO_2 ranges and the areas of the phase fields are

especially large group 9 and group 10 elements. We have added the details of the ab initio thermodynamics analysis in the SI and the following discussion in the revised manuscript:

(p.4) "To further evaluate the stability of the DMSCs against sintering and oxidation, we performed ab initio thermodynamics analysis following two previous studies.[33,34] We compared config A that we found this work (Figure 2a) with DMSCs from the literature and a typical 4+ configuration where the two metal atoms simply replace two Ce^{4+} on the surface (Figure S7). As shown in Figure S8, config A remain the most stable structure at 600 °C for a

range of O_2 pressures (pO_2). We further examined the stability of config A against sintering to large metal nanoparticles or oxidation to the 4+ state: stability diagrams in Figure S9 show that config A is stable in phase fields of certain temperature and pO_2 ranges and the areas of the phase fields are especially large group 9 and group 10 elements."

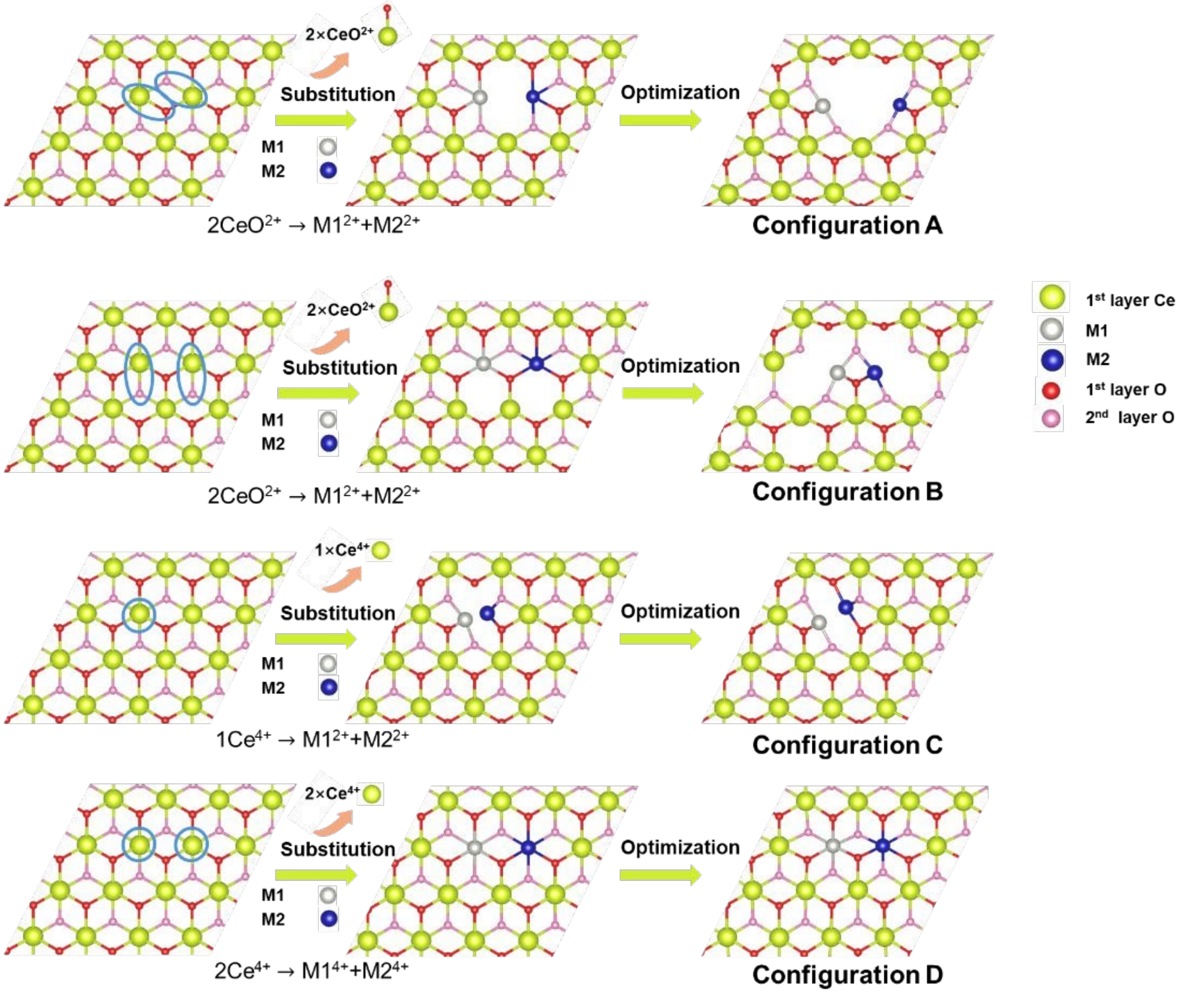

Figure S7. Four typical configurations of the DMSCs on $CeO_2(111)$: M1 and M2 in a 2+ nominal oxidation state in configs A, B, and C but 4+ in config D. Config A is the one used in the text that shows the linear-scaling-breaking behavior; config B is from ref. 31 in the text; config C is from ref. 32 in the text; config D is created from replacing two surface Ce^{4+} with $M1^{4+}$ and $M2^{4+}$.

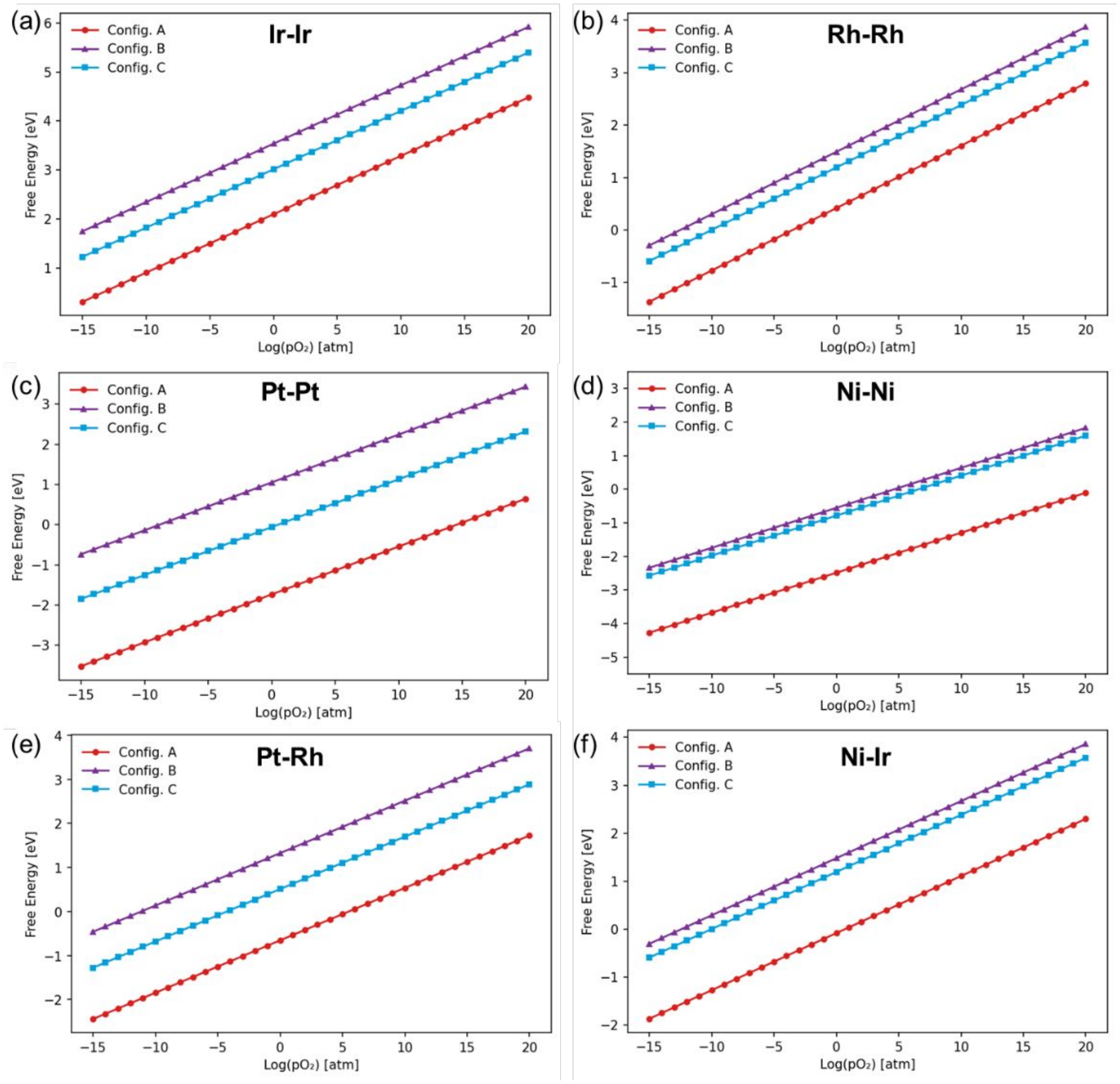

Figure S8. Relative stability of configurations A, B, and C for Ir-Ir, Rh-Rh, Pt-Pt, Ni-Ni, PtRh, Ni-Ir DMSCs at 600 ºC.

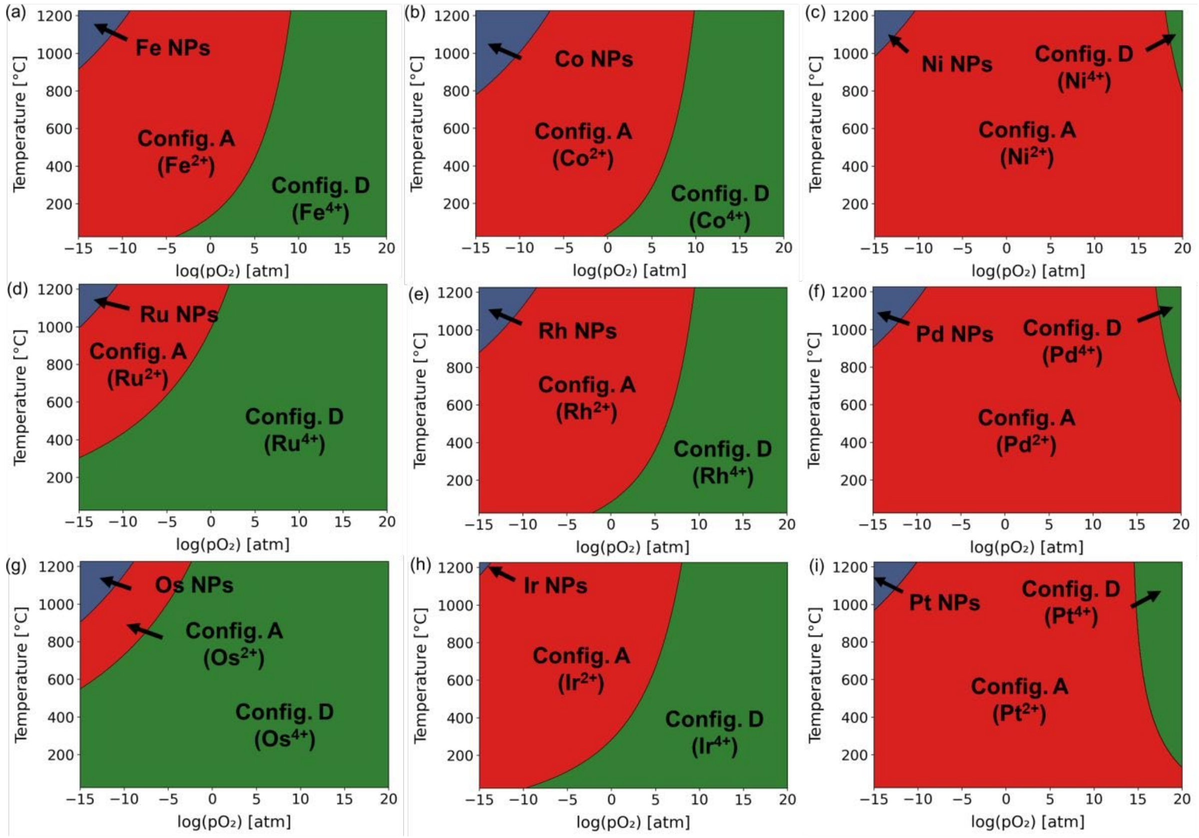

Figure S9. Stability diagrams of homonuclear DMSCs in config A vs large nanoparticles (NPs) and config D.

3. Minor: Coordinates of the relaxed and transition state structures (as well as the associated vibrational frequencies for TS saddle point confirmation) need to be reported in the SI.

[Author reply:] We have added the optimized initial state, final state structure for methyl coupling reactions, as well as transition state structure with the associated imaginary frequency to the SI (pp.S17-S23).

4. Minor: The AIMD settings need to be discussed in greater detail (missing information about the timestep, thermostat, equilibration time, etc.).

[Author reply:] We have added more computational details related to AIMD simulations in SI (p.S1) "AIMD simulations were performed within the canonical (NVT) ensemble using Nosé–Hoover thermostats,[1,2] with a time step of 1 femtosecond (fs) and a total simulation time of 10 picoseconds (ps)."
